# Cu(ii)-modified Mg–Al hydrotalcite/bentonite composites for adsorption and visible-light-driven photocatalytic degradation of Rhodamine B in textile wastewater

**DOI:** 10.1039/d5ra09642a

**Published:** 2026-03-23

**Authors:** Van Nhuong Vu, Thi Ha Thanh Pham, Thi Tu Anh Duong, Thi To Loan Nguyen, Truong Xuan Vuong

**Affiliations:** a Faculty of Chemistry, Thai Nguyen University of Education No. 20 Luong Ngoc Quyen Street Thai Nguyen City 24000 Vietnam; b Faculty of Natural Sciences and Technology, TNU-University of Science Tan Thinh Ward Thai Nguyen City 24000 Vietnam xuanvt@tnus.edu.vn

## Abstract

Dye pollution from textile effluents poses significant environmental challenges, necessitating efficient and sustainable treatment technologies. In this study, Cu(ii)-modified Mg–Al layered double hydroxide (5CuH) and its bentonite-supported composites were synthesized *via* a facile co-precipitation strategy and systematically characterized by XRD, SEM, BET, EDX, and UV-Vis DRS. Structural analysis confirmed successful Cu incorporation and uniform LDH dispersion on bentonite, resulting in narrowed band gaps (1.42–2.12 eV) and enhanced visible-light responsiveness. Under visible-light irradiation, 5CuH and 5CuH/Bent-2 achieved over 90% RhB degradation within 60 min at 50 ppm, accompanied by substantial mineralization. The 5CuH catalyst maintained high stability over four cycles with only 4.7% efficiency loss, while the composite showed moderate decline due to partial Cu^2+^ leaching but preserved structural integrity. Both materials effectively treated real textile wastewater, reducing COD to meet QCVN 40:2025/BTNMT discharge standards. This work demonstrates a synergistic clay-LDH design strategy that enhances charge separation and reactive oxygen species generation under visible light. Unlike adsorption-dominated systems, pollutant removal is primarily governed by photocatalytic oxidation, enabling efficient degradation and mineralization in complex wastewater matrices. The combination of low-cost raw materials, simple synthesis, and strong practical performance highlights the scalability and industrial potential of clay-supported LDH photocatalysts for sustainable textile wastewater remediation.

## Abstract 2

Dye pollution from textile effluents poses significant environmental challenges, necessitating efficient and sustainable treatment technologies.

## Graphical abstract

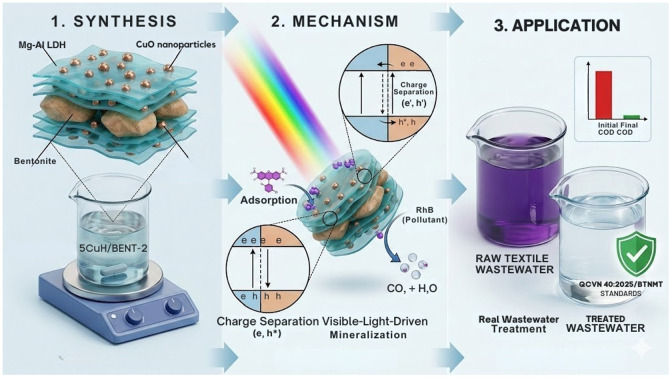

## 1. Introduction

Water pollution in Viet Nam has become increasingly serious, particularly in rivers and lakes located near textile dyeing villages and industrial zones.^1^ Textile effluents discharged without proper treatment contain high concentrations of synthetic dyes, surfactants, and auxiliary chemicals that persist in aquatic environments, reduce light penetration, inhibit photosynthesis, and pose risks to aquatic organisms and human health.^2^ Among these pollutants, textile dyes are especially problematic due to their chemical stability and toxicity.^3^

Rhodamine B (RhB), a cationic xanthene dye widely used in textile and printing industries, is frequently detected in dye-contaminated wastewater.^2^ It exhibits high solubility, intense coloration, and resistance to biodegradation. Prolonged exposure to RhB has been associated with toxic and potentially carcinogenic effects in aquatic systems.^2,4^

Adsorption and photocatalysis are two widely investigated strategies for dye removal.^5^ Adsorption enables rapid dye capture onto solid surfaces and is operationally simple and cost-effective;^6^ however, it predominantly transfers pollutants from water to an adsorbent phase without chemical transformation.^6^ Visible-light photocatalysis, in contrast, promotes oxidative degradation of organic molecules into smaller intermediates and, ideally, mineralized products such as CO_2_ and H_2_O.^7,8^ Integrating adsorption with photocatalysis within a single material architecture provides a rational route to couple surface enrichment with *in situ* catalytic oxidation, thereby increasing interfacial reaction probability and reducing mass-transfer limitations.^9,10^

Layered double hydroxides (LDHs), also known as hydrotalcites, have attracted considerable attention in environmental catalysis owing to their tunable metal composition, layered structure, and accessible redox-active sites.^11^ Mg–Al LDHs are among the most extensively studied systems due to their structural stability and relatively low cost.^9,12^ Partial substitution with transition metal ions such as Cu^2+^ or Ni^2+^ modifies the electronic structure, narrows the band gap, and introduces localized redox centers that extend visible-light absorption and influence charge carrier dynamics.^9,13^ For example, Vu *et al.* (2025) reported that Cu-substituted Mg–Al hydrotalcites achieved ∼94% visible-light degradation of Phenol Red within 120 min under 30 W LED irradiation.^9^

Despite these advantages, the intrinsically positively charged LDH layers restrict interfacial accessibility toward cationic dyes such as RhB due to electrostatic repulsion. This electrostatic constraint limits adsorption-driven surface concentration and reduces effective contact between dye molecules and catalytic redox sites.

Beyond conventional LDH systems, recent investigations have increasingly focused on electronic structure regulation and interfacial engineering to improve visible-light photocatalytic performance. Heterostructured LDH-based composites with tailored band alignment and directional charge-transfer pathways have been shown to suppress electron–hole recombination and enhance oxidative degradation efficiency.^14^ Transition-metal-modified hydrotalcite systems with improved dispersion and strengthened interfacial interaction demonstrated enhanced adsorption–photocatalytic coupling.^15^ Earlier studies further highlighted that compositional tuning and surface structural control govern carrier migration behavior and catalytic stability.^16^

Collectively, these studies underscore that photocatalytic performance in LDH-derived systems is not dictated solely by band-gap narrowing, but by interfacial charge redistribution, carrier separation efficiency, and spatial coupling between adsorption domains and redox-active centers.

To address the electrostatic limitation of LDHs toward cationic dyes, coupling LDH phases with negatively charged clay minerals represents a structurally coherent strategy. Bentonite, a naturally abundant and low-cost clay, possesses high specific surface area and strong cation-exchange capacity.^17,18^ Its negatively charged surface favors electrostatic adsorption of cationic dyes. However, bentonite lacks sufficient redox-active centers and sustained visible-light-driven charge separation.^19^ Although measurable optical absorption has been reported (apparent band gap 1.7–2.5 eV), partially attributed to trace metal species such as Fe^3+^ or Ti^4+^, pristine bentonite cannot maintain continuous reactive oxygen species (ROS) generation under visible irradiation. Dye removal by bentonite is therefore dominated by adsorption rather than photocatalytic oxidation.

Previous studies combining bentonite with photocatalytically active phases such as TiO_2_,^20,21^ g-C_3_N_4_,^21^ metal oxides,^22^ or LDHs^23^ demonstrated enhanced degradation efficiency by improving adsorption capacity and catalyst recoverability.^19–22^ For example, bentonite/TiO_2_ composites achieved up to 77% removal of direct red 80 and 100% removal of methylene blue under UV-assisted photocatalysis.^19^ Bouhent *et al.* (2024) reported Mg/Al-bentonite and Zn/Al-bentonite composites for Orange II removal with adsorption efficiencies of 99.92% and 74.48%, respectively.^23^

However, most reported systems primarily emphasize heterojunction formation or semiconductor band engineering. Systematic investigation of electrostatic coupling between positively charged Cu-modified LDH platelets and negatively charged clay substrates, particularly its influence on interfacial charge redistribution and carrier migration pathways, remains limited.

To contextualize recent advances, representative visible-light-driven RhB removal systems reported during 2024–2025 are summarized in Table 1.

**Table 1:** Representative recent studies (2024–2025) on visible-light-driven RhB removal

Study (year)	Material(s)	Mechanism	Key result	Conditions
g-C_3_N_4_/activated biochar (2025)^24^	g-C_3_N_4_ + activated biochar	Visible-light photocatalysis	∼98.7% Degradation (120 min)	LED irradiation; improved charge separation; >84% stability
BiOCl/Bi_2_O_3_/bentonite (2025)^25^	BiOCl + Bi_2_O_3_ + bentonite	Adsorption + photocatalysis	∼99.68% Degradation	Visible light; pseudo-first-order kinetics
Pd-In_2_O_3_/BiVO_4_ (2025)^26^	Pd-In_2_O_3_/BiVO_4_ heterostructure	Photocatalysis	Enhanced removal efficiency	Heterojunction-assisted charge transfer
In-doped ZnO (2024)^27^	In-doped ZnO nanoparticles	Photocatalysis	∼93% Degradation	Band-gap narrowing
Mn_3_O_4_/ZnO/AC (2025)^28^	Mn_3_O_4_/ZnO/activated carbon	Photocatalysis + mineralization	∼95.85% Removal; 80.56% mineralization	Carbon-enhanced interfacial charge transfer
This work (2026)	Cu-modified Mg–Al hydrotalcite/bentonite	Adsorption + visible-light photocatalysis + mineralization	Significant COD reduction in real textile wastewater	Integrated adsorption–photocatalysis platform

As summarized in Table 1, state-of-the-art systems predominantly rely on heterojunction engineering, doping strategies, or carbon-supported architectures to suppress recombination and enhance visible-light utilization.^24–28^ While high decolorization efficiencies are frequently reported, systematic assessment of mineralization performance and validation in complex real textile wastewater matrices remain comparatively limited. In addition, the role of electrostatic-driven interfacial coupling between LDH platelets and clay substrates has not been thoroughly examined from a charge-dynamics perspective.

Therefore, in this study:

(i) Cu(ii)-modified Mg–Al hydrotalcite (5CuH, Mg : Cu : Al = 2 : 5 : 3) was synthesized *via* coprecipitation and integrated with bentonite at different mass ratios. The selected metal ratio was optimized based on prior benchmarks demonstrating superior visible-light activity.^9^

(ii) Structural, morphological, textural, and optical properties were characterized (XRD, SEM, BET, UV-Vis DRS, EDX) to elucidate structure–interfacial coupling–charge transport relationships.

(iii) Adsorption behavior, visible-light photocatalytic degradation of RhB, and COD reduction in real textile wastewater were systematically evaluated.

This work establishes a structure–interfacial electrostatic coupling–photocatalytic performance correlation, demonstrating how Cu^2+^ incorporation and clay-assisted charge redistribution cooperatively regulate adsorption enrichment, carrier separation, and oxidative mineralization under visible-light irradiation. The resulting composite platform provides a mechanistically informed and scalable strategy for sustainable treatment of cationic dye-contaminated wastewater.

## 2. Materials and methods

### 2.1. Materials

All reagents were of analytical grade and used without further purification. Magnesium nitrate hexahydrate, aluminum nitrate nonahydrate, copper(ii) nitrate trihydrate, sodium carbonate, and EDTA-2Na were purchased from Merck (Germany). Sodium hydroxide, hydrochloric acid, sulfuric acid, potassium dichromate, silver sulfate, mercury(ii) sulfate, potassium iodide, silver nitrate, potassium hydrogen phthalate, isopropyl alcohol, ascorbic acid, and Rhodamine B were obtained from Xilong Scientific (China). Natural bentonite clay was supplied from India. Deionized water was used throughout all syntheses and experiments.

### 2.2. Synthesis of Cu-modified hydrotalcite (5CuH)

**Fig. 1:**
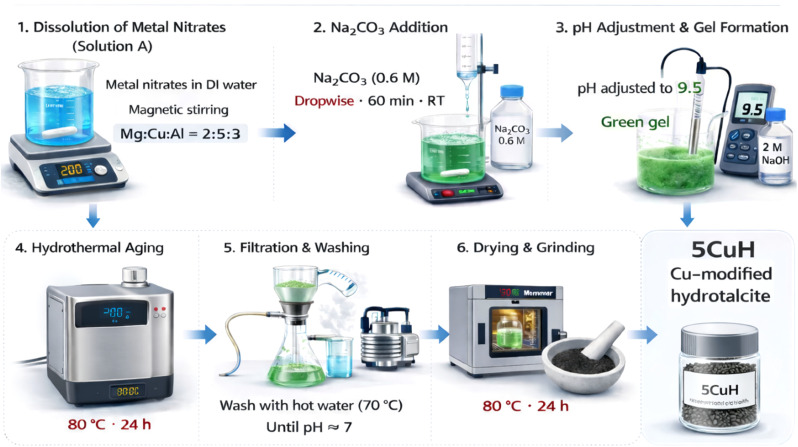
Schematic representation of the synthesis process of 5CuH.

Cu-modified Mg–Al hydrotalcite (5CuH) was prepared *via* a co-precipitation method. In a typical synthesis, 11.25 g of Al(NO_3_)_3_·9H_2_O, 5.15 g of Mg(NO_3_)_2_·6H_2_O, and 12.10 g of Cu(NO_3_)_2_·3H_2_O were dissolved in 100 mL of deionised water in a 500 mL beaker under magnetic stirring to obtain solution A. The overall molar ratio of Mg : Cu : Al : CO_3_^2−^ was maintained at 2 : 5 : 3 : 1.5. Subsequently, 25 mL of 0.6 M Na_2_CO_3_ solution (1.59 g) was added dropwise to solution A under continuous stirring for 60 min at room temperature. The pH of the suspension was then adjusted to 9.5 by the careful addition of 2 M NaOH, resulting in the formation of a green gel. The gel was stirred for an additional 60 min at room temperature, followed by hydrothermal aging at 80 °C for 24 h in a Memmert oven. The precipitate was recovered by filtration and washed repeatedly with hot water (70 °C) until the filtrate reached neutral pH (≈7.0). The solid was dried at 80 °C for 24 h and subsequently ground in an agate mortar to yield a fine grey-black powder, denoted as 5CuH.^9,29^ The synthesis process of the 5CuH material is schematically illustrated in Fig. 1.

### 2.3. Synthesis of Cu-modified hydrotalcite/bentonite composites (5CuH/Bent)

Bentonite-based composites containing Cu-modified Mg–Al hydrotalcite were synthesised following procedures adapted from Mohammed *et al.* (2023)^30^ and (2024).^23^ Three beakers (500 mL) were each loaded with 3.00 g of bentonite and 60 mL of deionised water. The suspensions were left to swell for 24 h to allow full hydration of the bentonite layers. Subsequently, 100 mL of precursor solution A, containing Al(NO_3_)_3_·9H_2_O, Mg(NO_3_)_2_·6H_2_O, and Cu(NO_3_)_2_·3H_2_O in a molar ratio of Mg : Cu : Al : CO_3_^2−^ = 2 : 5 : 3 : 1.5, was added dropwise to each swollen bentonite suspension. The exact nitrate salt amounts used in each synthesis are listed in Table 2.

**Table 2:** Sample codes and precursor quantities used for the synthesis of bentonite/Cu–Mg–Al hydrotalcite composites^a^

Sample code	Precursor salts (g)		Bentonite (Bent) (g)	Bentonite content (wt%)
5CuH/Bent-1	Mg(NO_3_)_2_·6H_2_O	1.707	3.000	23.04%
	Cu(NO_3_)_2_·6H_2_O	4.033		
	Al(NO_3_)_3_·9H_2_O	3.750		
	Na_2_CO_3_	0.530		
5CuH/Bent-2	Mg(NO_3_)_2_·6H_2_O	3.413	3.000	13.02%
	Cu(NO_3_)_2_·6H_2_O	8.067		
	Al(NO_3_)_3_·9H_2_O	7.500		
	Na_2_CO_3_	1.060		
5CuH/Bent-3	Mg(NO_3_)_2_·6H_2_O	5.120	3.000	9.07%
	Cu(NO_3_)_2_·6H_2_O	12.100		
	Al(NO_3_)_3_·9H_2_O	11.250		
	Na_2_CO_3_	1.590		

a Bentonite content is expressed as a percentage of the total solid mass, calculated following ref. 31.

The mixtures were stirred vigorously at 500 rpm for 60 min, after which 25 mL of 0.6 M Na_2_CO_3_ solution was introduced dropwise. Stirring was continued for an additional 60 min, followed by pH adjustment to 9.5 using 2 M NaOH to induce gel formation. The resulting gels were stirred for another 60 min at room temperature, then aged at 80 °C for 24 h in an oven. The products were recovered by filtration and washed repeatedly with hot water (70 °C) until neutral pH (≈7.0). The solids were dried at 80 °C for 24 h, ground in an agate mortar, and labelled 5CuH/Bent-1, 5CuH/Bent-2, and 5CuH/Bent-3.

**Fig. 2:**
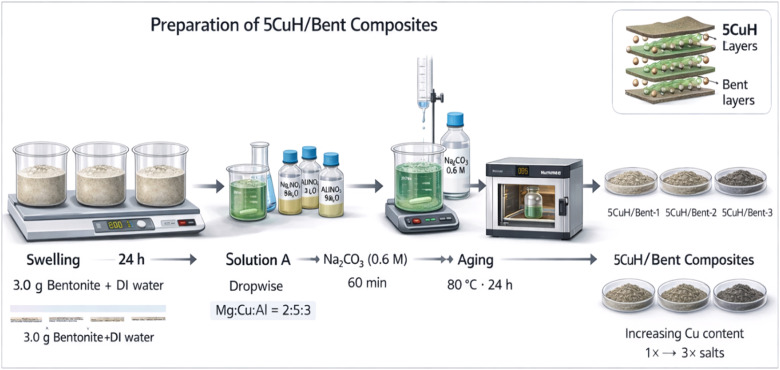
Schematic illustration of the synthesis process of the 5CuH/Bent composite material.

In these composites, the quantities of Mg(NO_3_)_2_·6H_2_O, Cu(NO_3_)_2_·3H_2_O, Al(NO_3_)_3_·9H_2_O, and Na_2_CO_3_ were systematically varied: the salt masses used for 5CuH/Bent-2 and 5CuH/Bent-3 were two- and three-fold higher, respectively, compared with those used in 5CuH/Bent-1. The synthesis procedure of the 5CuH/Bent composite is illustrated in Fig. 2.

### 2.4. Preparation of Rhodamine B standard solutions and UV-Vis calibration curve

A stock solution of Rhodamine B (RhB, 500 ppm) was prepared by accurately weighing 0.500 g of RhB and dissolving it in double-distilled water to a final volume of 1000 mL (denoted as solution A). From this stock, 100 mL was diluted fivefold to obtain an intermediate solution of 100 ppm (solution B). A series of working solutions with RhB concentrations of 0.5, 1.0, 2.0, 4.0, 6.0, 8.0, 10.0, 12.0, and 15.0 ppm was subsequently prepared from solution B to a final volume of 100 mL each. The UV-Vis absorption spectra of these solutions were recorded in the wavelength range of 200–800 nm using a UV-1700 spectrophotometer. The maximum absorption band of RhB at 553 nm was selected for quantitative analysis, and a calibration curve was constructed accordingly (see Table S1 and Fig. S1 in the SI).

### 2.5. Characterization techniques

The structural, morphological, and physicochemical properties of the synthesized materials were investigated using a range of complementary techniques. Powder X-ray diffraction (XRD) patterns were recorded on a MiniFlex600 diffractometer (Rigaku, Japan) to determine the crystalline phase composition and average crystallite size. The surface morphology and layered platelet structure were examined by scanning electron microscopy (SEM) using a Hitachi S-4800 instrument (Japan). Elemental composition was analyzed by energy-dispersive X-ray spectroscopy (EDS) with a HORIBA 7593-H system (Kyoto, Japan). Fourier-transform infrared (FT-IR) spectra were collected on a Spectrum Two DTGS spectrometer (PerkinElmer, Waltham, MA, USA) to identify functional groups and bonding environments. Textural properties, including nitrogen adsorption–desorption isotherms, specific surface area, pore volume, and pore size distribution, were obtained using a TriStar II 3020 analyzer (Micromeritics, USA). The optical absorption edge and band-gap energy of the samples were evaluated by UV-Vis diffuse reflectance spectroscopy (UV-Vis DRS) using a HITACHI U-4100 spectrophotometer (Tokyo, Japan).

### 2.6. Adsorption experiments

#### 2.6.1. Adsorption of Rhodamine B in the dark

The adsorption performance of the synthesized materials toward Rhodamine B (RhB) was evaluated under dark conditions to exclude any photocatalytic contribution. In a typical experiment, 0.20 g of catalyst was dispersed in 250 mL of RhB solution (50 ppm) contained in a 500 mL glass beaker. The beaker was wrapped in black polyethylene film to prevent light penetration and magnetically stirred at room temperature (25–30 °C) for 30 min to establish adsorption–desorption equilibrium between the dye molecules and the material surface. Aliquots were withdrawn at equilibrium, centrifuged to remove suspended solids, and analyzed by UV-Vis spectrophotometry to determine the residual RhB concentration. The adsorption efficiency (%) was calculated as:1
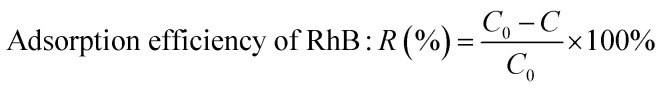
where *C*_0_ is the initial concentration of RhB and *C* is the concentration after equilibrium was reached.

The adsorption behavior of five samples, bentonite (Bent), Cu-modified hydrotalcite (5CuH), and the composite materials 5CuH/Bent-1, 5CuH/Bent-2, and 5CuH/Bent-3, was investigated simultaneously under identical conditions. From the obtained results, the RhB adsorption efficiency (%) and the equilibrium adsorption time for each material were evaluated and compared.

#### 2.6.2. Evaluation of the photocatalytic dye degradation activity of the material under visible light

##### 2.6.2.1 Effect of irradiation time

For photocatalytic experiments, 0.2 g of catalyst was dispersed in a 500 mL beaker containing 250 mL of Rhodamine B (RhB) solution (50 ppm). The beaker was wrapped in black polyethylene film and stirred magnetically at room temperature for 30 min to establish adsorption–desorption equilibrium. Aliquots were collected to evaluate the adsorption efficiency as described in Section 2.6.1. This procedure was performed simultaneously for all five investigated catalysts.

After the adsorption equilibrium was reached, 1.2 mL of 30% H_2_O_2_ was added to each suspension. The mixtures were then irradiated with a 30 W LED lamp while stirring continuously at 500 rpm (a 30 W Philips LED lamp, 6500 K, luminous flux of 2550 Lm, with a maximum emission wavelength at 464 nm). The reaction process was carried out as described in Fig. S2 (see SI). At fixed time intervals (every 30 min), samples were withdrawn, centrifuged, and analyzed by UV-Vis spectroscopy (UV-1700, 200–800 nm) to determine the absorbance of RhB at each interval. The RhB removal efficiency was calculated using eqn (2):2
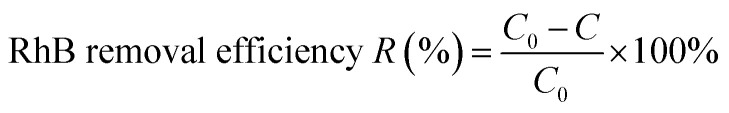
where *C*_0_ is the initial RhB concentration and *C* is the concentration at survey time.

From the obtained results, the influence of irradiation time on the degradation efficiency of 50 ppm RhB by the tested materials can be evaluated. Furthermore, based on these findings, the material exhibiting the highest RhB removal performance among the five samples investigated can be identified. To determine the photocatalytic degradation efficiency of RhB, one of the following equations can be applied:3
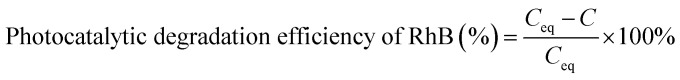
where *C*_eq_ is the RhB concentration at equilibrium, and *C* is the RhB concentration at the selected irradiation time.

Alternatively, the photocatalytic degradation efficiency of RhB (%) can be calculated as:

4 Photocatalytic degradation efficiency of RhB = total removal efficiency of RhB (%) − adsorption efficiency of RhB at equilibrium (%)

##### 2.6.2.2 Effect of RhB concentration

Two materials exhibiting superior catalytic activity, namely 5CuH and 5CuH/Bent-2, representing hydrotalcite and composite catalysts, were selected to investigate the influence of RhB concentration on photocatalytic degradation under LED irradiation. For each experiment, 0.2 g of catalyst was added to a 500 mL glass beaker containing 250 mL RhB solution of varying concentrations (50, 75, and 100 ppm). The suspensions were wrapped in black plastic to exclude light and magnetically stirred at room temperature for 30 min to establish adsorption equilibrium. Subsequently, 1.2 mL of 30% H_2_O_2_ was introduced, and the mixtures were irradiated with a 30 W LED lamp while stirring at 500 rpm. After every 30-minute interval, aliquots were withdrawn, centrifuged to remove solids, and the supernatants were analyzed. For RhB concentrations of 75 and 100 ppm, the samples were diluted tenfold prior to measurement. All experiments were conducted in triplicate under identical conditions. Based on the obtained results, the effect of RhB concentration on the photocatalytic activity of the materials was evaluated.

##### 2.6.2.3 Investigation of the role of light on the photocatalytic activity

Two representative materials, 5CuH and 5CuH/Bent-2, were employed to evaluate their performance in the degradation of 75 ppm RhB under two different conditions: (i) with LED irradiation and (ii) in the absence of light (dark conditions), in order to assess the role of a 30 W LED source. The experimental procedures followed those described in section “Effect of RhB concentration”. For the dark condition, all room lights were switched off and the reaction vessels were completely wrapped in black polyethylene to prevent any external light from entering the system.

##### 2.6.2.4 Effect of solution pH

The pH of 100 ppm RhB solutions was adjusted using 0.5 N NaOH or 0.5 N HCl to the desired values, ensuring that the final volume change was negligible. The initial pH of the 100 ppm RhB solution was 3.9. For this study, the pH was adjusted to 3.0, 6.0, 8.0, 10.0, and 12.0. Two representative materials, 5CuH and 5CuH/Bent-2, were selected to evaluate the influence of solution pH on their catalytic activity. Following 30 min of stirring in the dark to achieve adsorption equilibrium at each pH, the experimental procedure was carried out as described in section “Effect of RhB concentration”. Based on the obtained results, the effect of pH on the RhB removal efficiency of 5CuH and 5CuH/Bent-2 was assessed.

##### 2.6.2.5 Investigation of radical trapping and proposed RhB degradation mechanism

The advanced oxidation process (AOP) is associated with the generation of photoinduced electron–hole (e^−^–h^+^) pairs, reactive oxygen species (ROS) such as ˙OH, O_2_˙^−^, HOO˙, singlet oxygen (^1^O_2_), and reactive sulfate radicals (SO_4_˙^−^, SO_5_˙^−^). These species can participate in the oxidation or reduction of organic pollutants in aqueous environments, leading to the formation of intermediates and ultimately CO_2_ and H_2_O. To identify the dominant reactive species involved in RhB degradation (100 ppm), selective scavengers were introduced, including AgNO_3_ (1 mM, e^−^ scavenger), EDTA-2Na (1 mM, h^+^ scavenger), ascorbic acid (1 mM, O_2_˙^−^ scavenger), and isopropyl alcohol (10 mM, ˙OH scavenger), following previously reported protocols.^32–34^ Two representative samples, 5CuH and 5CuH/Bent-2, were tested. After the adsorption equilibrium was reached, 1.2 mL of 30% H_2_O_2_ was added, followed by 2.0 mL of the scavenger solution. The experiments were carried out under the same conditions as described in section “Effect of RhB concentration”. Based on the inhibition effects observed with different scavengers, the key reactive species responsible for RhB degradation could be identified, allowing for the proposal of a degradation mechanism for the studied materials.

### 2.7. Reusability and stability of the composites

The determination of the stability of two materials, with different compositions (5CuH and 5CuH/Bent-2), was evaluated for reuse by means of adsorption/desorption equilibrium followed by LED lamp irradiation of the reaction mixture under dark conditions. Reproduced, the initial reaction mixture consisted of 0.200 g catalyst, 250 mL of a 100 ppm solution of RhB, and 1.2 mL of 30% hydrogen peroxide (H_2_O_2_) solution in addition to the catalyst. To reach adsorption/desorption equilibrium, the mixture was stirred in the dark for 30 minutes prior to the addition of H_2_O_2_. Once the reaction mixture was reacted under UV light for 180 minutes using 30 W LED lamp, approximately every 60 minutes, approximately 7–8 mL (to allow for centrifugation and determination of the absorbance of RhB) was withdrawn from the reaction mixture, after which the catalyst was separated by centrifuging and diluted tenfold before analysis. The time at which the derivative Cu^2+^ ions leached from the material into solution was determined using a fivefold dilution of the filtered leachate before the analysis for each of the sampling periods.

All of the suspension remaining after the termination of the photocatalytic test of both catalysts was made into a single combined suspension, filtered, and thoroughly washed on the Buchner funnel (under process conditions) to collect all of the materials in distilled water at 70 °C until they produced a light pink filtrate. All recovered materials were subsequently dried in an oven overnight at 80 °C and were subjected to the same process for further photocatalytic reactions. The use and reuse of both catalysts were repeated using the same experimental conditions, with comparisons and discussion made on the durability of the individual catalysts, along with the change in their catalytic activity as they were successively reused for photocatalytic reactions.

### 2.8. Real wastewater application

Two representative materials, 5CuH and 5CuH/Bent-2, were employed to investigate their ability to treat diluted mat-dyeing wastewater. Before the experiments, the raw wastewater was diluted tenfold (initial pH as 5.08). Each reaction system contained 0.2 g of catalyst and 1.2 mL of 30% H_2_O_2_. The suspensions were first stirred in the dark for 60 min to establish adsorption–desorption equilibrium, followed by visible-light irradiation. Aliquots were withdrawn at 30-minute intervals, centrifuged, and diluted fivefold before recording the UV-Vis absorption spectra. The treatment efficiency for the dyes present in the wastewater was calculated using eqn (5):5
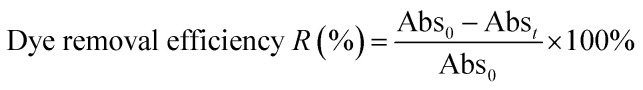
where Abs_0_ is the initial absorbance of the wastewater solution at its maximum absorption wavelength, and Abs_*t*_ is the absorbance at a given irradiation time *t*.

### 2.9. Mineralization capacity of the materials

#### 2.9.1. Mineralization of RhB (100 ppm) at pH 6.0

The composite sample 5CuH/Bent-2 was selected to investigate the mineralization efficiency of RhB (100 ppm) over the treatment period. At each sampling interval, aliquots were centrifuged to remove suspended solids and subsequently analyzed for COD. To eliminate residual H_2_O_2_, 2.5 mL of the RhB solution was transferred into COD digestion cuvettes, adjusted to pH 10.0 with 3–4 drops of 2 N NaOH, and refluxed at 100 °C for 30 min to ensure complete decomposition of H_2_O_2_. After cooling, 1.5 mL of the oxidizing reagent mixture (10.216 g K_2_Cr_2_O_7_ + 33.3 g HgSO_4_ + 167 mL concentrated H_2_SO_4_, diluted to 1 L) and 3.5 mL of the acid catalyst solution (5.5 g Ag_2_SO_4_ in 500 mL concentrated H_2_SO_4_) were added sequentially. The cuvettes were then refluxed at 150 °C for 2 h, and the absorbance of the digested samples was measured at 600 nm using a UV-1700 spectrophotometer. The COD values obtained were used to calculate the degree of RhB mineralization according to eqn (6).

#### 2.9.2. Mineralization of dyeing wastewater

The materials 5CuH and 5CuH/Bent-2 were also employed to evaluate the mineralization of organic pollutants present in diluted mat-dyeing wastewater during the treatment process. COD values were determined at fixed treatment intervals to assess the degree of mineralization. These experiments were conducted in parallel with the dye degradation tests described in Section 2.6. At each 1 h interval, samples were withdrawn, centrifuged to remove solids, and analyzed without dilution. Subsequent COD determination followed the same procedure described in Section 2.6. The extent of wastewater mineralization achieved by the tested materials was calculated based on COD reduction using eqn (6).6
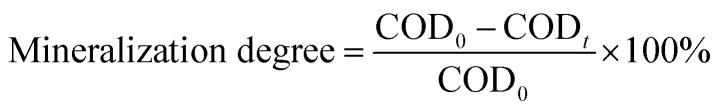
where COD_0_ was the COD value at the initial time and COD_*t*_ was the COD value at the sampling time.

## 3. Results and discussion

### 3.1. Structural and morphological characterization

#### 3.1.1. X-ray diffraction analysis of the synthesized materials

**Fig. 3:**
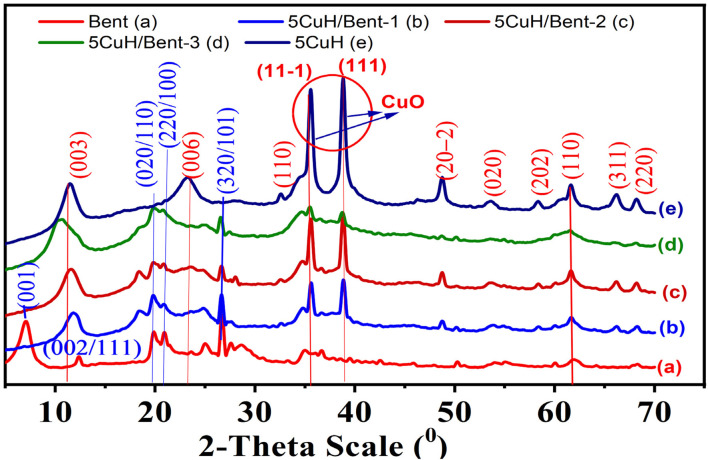
XRD patterns of the synthesized materials and pristine bentonite: Bent (a), 5CuH/Bent-1 (b), 5CuH/Bent-2 (c), 5CuH/Bent-3 (d), and 5CuH (e).

The XRD pattern of the pristine bentonite sample (Fig. 3) displays characteristic diffraction peaks at 2*θ* = 7.17° (plane (001)), 12.40° (planes (111) or (002)), 19.84° (planes (020) or (110)), 20.89° (planes (220), (021), or (100)), 24.97° (planes (311) or (004)), 26.72° (planes (320) or (101)), and 36.63° (plane (110)). These reflections are consistent with previously reported data.^31,35,36^ In particular, the Na-bentonite (B-Na^+^) described in a previous study^35^ exhibited a basal spacing of *d*_(001) = 12.47 Å at 2*θ* = 7.16°, which closely matched the result obtained in this study (*d*_(001) = 12.32 Å at 2*θ* = 7.17°). The montmorillonite phase was identified by reflections at (001), (110), (220), (311), and (320), as indexed in the DB card number 1540237. Kaolinite is characterized by peaks at (002), (020), (021), and (004) (DB card number 1011045). Quartz is present with reflections at (110, 2*θ* = 36.63°), (102, 2*θ* = 39.56°), and (200, 2*θ* = 42.52°) according to DB card number 9012600. Thus, the characteristic peaks of montmorillonite, kaolinite, and quartz phases were clearly observed in the XRD pattern of the bentonite sample (curve a in Fig. 3).

The XRD pattern of the 5CuH sample exhibited the characteristic reflections of a hydrotalcite-like layered structure at 2*θ* = 11.53° ((003) plane), 23.14° ((006)), 38.77° ((015)), 46.52° ((018)), and 61.59° ((113)), in accordance with DB card number 9009272.^37,38^ The basal spacing was calculated as *d*_003_ = 7.667 Å, with corresponding lattice parameters *a* = 3.01 Å and *c* = 23.01 Å (where a represents the cation–cation distance in the brucite-like layer and *c* is the layer thickness, defined as *a* = 2*d*_110_ and *c* = 3*d*_003_). These values confirmed that the 5CuH sample retained a hydrotalcite-like structure with CO_3_^2−^ anions located in the interlayer region.^9,29,39^

In addition, diffraction peaks assigned to tenorite-type CuO were also observed at 2*θ* = 35.55° ((11−1) plane), 38.77° ((111)), 48.72° ((20−2)), 53.56° ((020)), 58.29° ((202)), 61.59° ((113)), 66.07° ((311)), and 68.15° ((220)), consistent with DB card number 9014580. These CuO reflections closely match those reported for CuO/MgAl-LDHs by other authors.^37,40^ A distinct feature of 5CuH, when compared to Cu^2+^-substituted ZnAl- and MgAl-hydrotalcites described in previous studies,^9,29,39^ was the appearance of crystalline CuO. This was attributed to the aging temperature of 80 °C applied during synthesis, at which partial decomposition of Cu(OH)_2_ occurs, leading to CuO formation. In contrast, materials synthesized at lower temperatures (*e.g.*, 70 °C) generally retained the hydroxide phase without CuO crystallization. The formation of CuO at 80 °C was in agreement with the report of Zhang *et al.* (2022),^41^ who demonstrated that higher aging temperatures markedly influenced the crystallinity of LDHs. Specifically, aging at temperatures ≥85 °C leaded to the decomposition of Cu(OH)_2_ and Co(OH)_2_, resulting in the formation of crystalline CuO and Co_3_O_4_ phases, which was unfavorable for the direct synthesis of Co–Cu LDHs.

The XRD patterns of the composite samples 5CuH/Bent-1, 5CuH/Bent-2, and 5CuH/Bent-3 revealed the characteristic reflections associated with the crystalline phases of bentonite, with the exception of the basal reflection at 2*θ* = 7.17° (*d*_001_), which was absent in all three composites. This absence could be explained by two factors: (i) the relatively lower bentonite content compared with hydrotalcite 5CuH in the composite formulations (see bentonite weight percentages in Table 1), and (ii) the intercalation of bentonite platelets within the layered structure of hydrotalcite 5CuH.^31,35,42^ When bentonite was dispersed in water for 24 h, swelling occurs, increasing the interlayer spacing and facilitating the intercalation of hydrated cations (Cu^2+^, Mg^2+^, Al^3+^) into the interlayer galleries. As a result, the obtained composites exhibited structural features derived from both bentonite and hydrotalcite 5CuH. Furthermore, the structural delamination of bentonite during the synthesis process allowed the hydrotalcite layers to be integrated within the bentonite galleries rather than being merely deposited on the bentonite surface.^35^

However, the XRD patterns of the composites 5CuH/Bent-1 and 5CuH/Bent-2 exhibited the characteristic reflections of both 5CuH and bentonite, with sharp and intense peaks, particularly in the case of 5CuH/Bent-2. In contrast, the 5CuH/Bent-3 sample showed pronounced differences compared with pure 5CuH and bentonite: (i) the basal reflection associated with the hydrotalcite layered structure shifts from 2*θ* = 11.53° in 5CuH to 10.45° (003 plane), indicating a significant collapse of the hydrotalcite lamellar structure; and (ii) the intensities of the characteristic reflections of hydrotalcite, bentonite, and CuO phases are all markedly reduced in this sample. Therefore, the XRD analysis suggested that among the three synthesized composites, 5CuH/Bent-2 retained the fundamental structural features of both hydrotalcite and bentonite most effectively.

Table 3 summarizes the fundamental structural parameters of the five samples. The average crystallite size was calculated using the Scherrer equation.^30^

7 *D* = 0.89*λ*/*b* cos *θ*

where *D* is the average crystallite size, *λ* is the wavelength of the incident X-ray (*λ* = 0.154 nm), *θ* is the Bragg angle (half of the 2*θ* value, expressed in radians), and *β* is the full width at half maximum (FWHM) of the diffraction peak (in radians).

**Table 3:** Fundamental structural parameters of the synthesized materials^a^

Sample	*d*_(001)_ (Å)	*d*_(003)_ (Å)	*d*_(110)_ (Å)	Lattice parameters *a*, *c* of hydrotalcite (Å)	Crystallite size of bentonite (Å)	Crystallite size of hydrotalcite (Å)	Crystallite size of CuO (Å)
Bentonite	12.32				60.88		
5CuH	—	7.67	1.50	3.01; 23.01	—	49.71	196.13
5CuH/Bent-1	—	7.43	1.50	3.00; 22.29	—	51.52	272.31
5CuH/Bent-2	—	7.62	1.50	3.01; 22.86	—	46.37	244.60
5CuH/Bent-3	—	**8.46**	1.50	3.01; **25.38**	—	31.0	—

a The average crystallite sizes were calculated using the Scherrer equation with Cu Kα radiation (*λ* = 0.154 nm) and “—” indicates that the parameter could not be determined.

#### 3.1.2. Scanning electron microscopy (SEM) of the materials

**Fig. 4:**
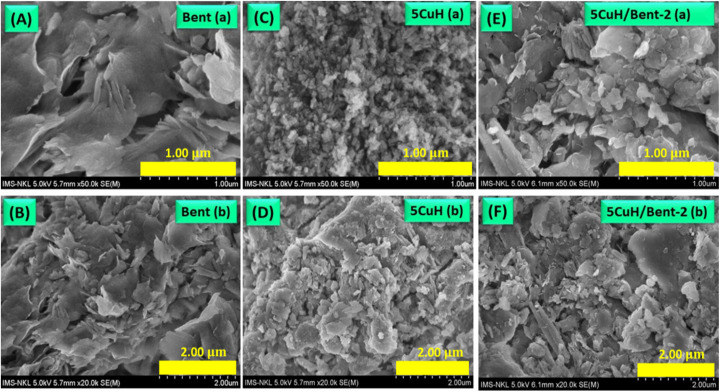
SEM micrographs of Bent (A and B), 5CuH (C and D), and 5CuH/Bent-2 (E and F), obtained at magnifications of 1.00 µm and 2.00 µm.

Three representative samples, including bentonite (Bent), 5CuH, and 5CuH/Bent-2, were selected for SEM imaging in order to examine their layered morphology and particle size characteristics (Fig. 4A–F).

Fig. 4A and B showed SEM images of Bent recorded at two different magnifications. The Bent sample exhibited stacked clay platelets with highly irregular sizes: while some platelets measured only a few tens of nanometers, others reached several hundred nanometers. In contrast, the 5CuH sample displayed much smaller platelets compared to Bent, with dimensions of only several tens of nanometers, and a more uniform distribution (Fig. 4C and D). When bentonite was combined with hydrotalcite (5CuH), the resulting composite revealed an intermixture of bentonite and hydrotalcite platelets (Fig. 4E and F). Importantly, the bentonite platelets were markedly reduced in size relative to the pristine material, while hydrotalcite platelets appeared interspersed among them. These morphological observations were consistent with the XRD results discussed above.

#### 3.1.3. Energy-dispersive X-ray spectroscopy (EDS) of the materials

**Fig. 5:**
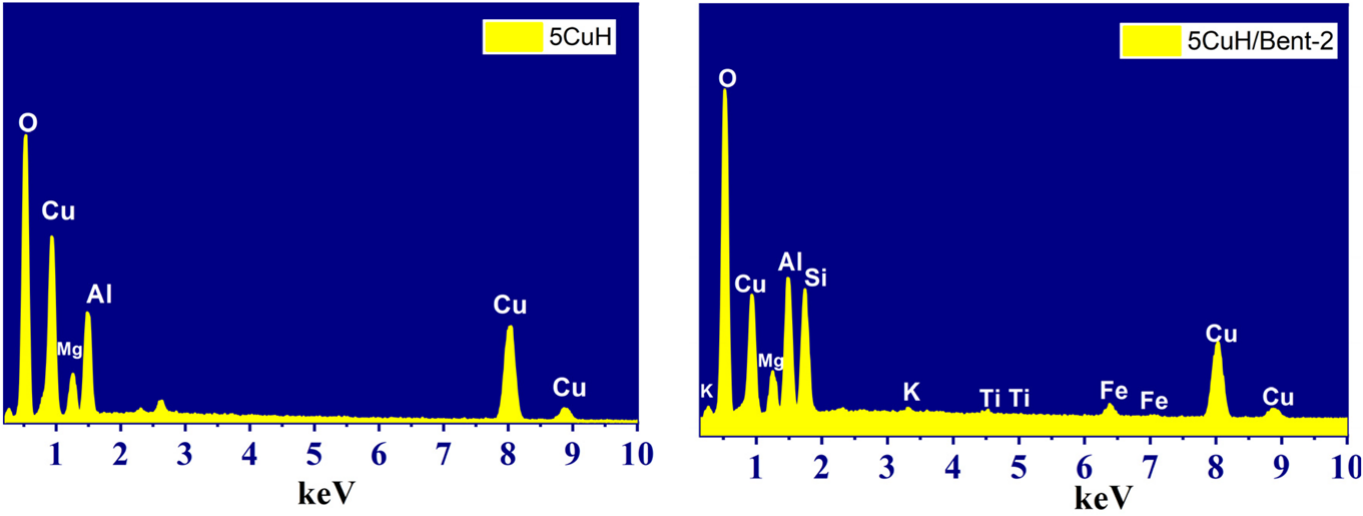
EDS spectra of the representative samples 5CuH and 5CuH/Bent-2.

Two representative samples, 5CuH and 5CuH/Bent-2, were subjected to EDS analysis to determine their elemental composition. The EDS spectrum of 5CuH revealed the presence of five elements: O, Mg, Al, Cu, and C (Fig. 5 and Table S1 (in SI)). In contrast, the 5CuH/Bent-2 composite exhibited a more complex composition, containing eight elements: K, Cu, Mg, Al, Si, Ti, Fe, and O (Fig. 5 and Table S2 (in SI)).

The molar ratio of Cu : Mg : Al in the 5CuH sample was calculated to be 13.31 : 5.52 : 9.46, corresponding approximately to 5.18 : 2.0 : 3.43. This ratio was in close agreement with the theoretical metal ratio used for the synthesis of the material (5.0 : 2.0 : 3.0). In the composite sample 5CuH/Bent-2, the Cu : Mg : Al molar ratio was determined as 9.37 : 3.92 : 8.46, equivalent to 4.75 : 2.0 : 4.32, which deviated slightly from the initial theoretical ratio due to the incorporation of bentonite. Furthermore, the EDS spectrum of 5CuH/Bent-2 revealed the presence of additional elements, namely Si (6.96 at%), K (0.18 at%), Ti (0.14 at%), and Fe (0.81 at% (Table S2 (SI))). These elements originate from the parent bentonite. Since calcium was absent in the composition, the Indian bentonite employed in this study can be classified as an alkali-metal bentonite containing K.

#### 3.1.4. N_2_ adsorption–desorption isotherms (BET) of the materials

**Fig. 6:**
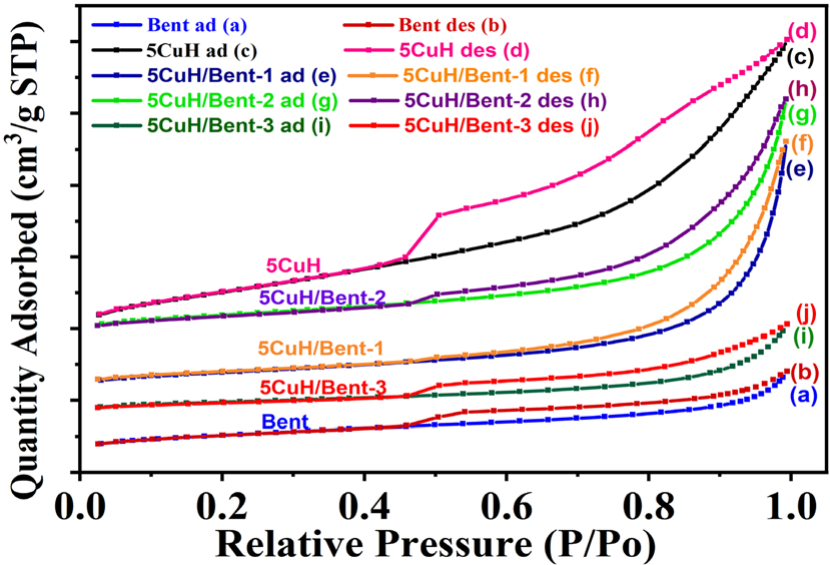
N_2_ adsorption–desorption (BET) isotherms of the synthesized materials at 77 K. The isotherms are arranged from the lowest to the highest adsorbed volume (at a relative pressure of *P*/*P*_0_ = 0.4) as follows: Bent, 5CuH/Bent-3, 5CuH/Bent-1, 5CuH/Bent-2, and 5CuH. (Bent: adsorption (a) and desorption (b) 5CuH: adsorption (c) and desorption (d) 5CuH/Bent-1: adsorption (e) and desorption (f) 5CuH/Bent-2: adsorption (g) and desorption (h) 5CuH/Bent-3: adsorption (i) and desorption (j)).

The N_2_ adsorption–desorption isotherms of the five materials are presented in Fig. 6. All samples exhibited a hysteresis loop in the relative pressure range of 0.45–1.0, indicating type IV isotherms with H3-type hysteresis according to IUPAC classification, characteristic of mesoporous, plate-like structures.^42^ Bentonite and 5CuH/Bent-3 displayed relatively narrow hysteresis loops, whereas the remaining three samples, 5CuH/Bent-1, 5CuH/Bent-2, and 5CuH, showed broader loops, with 5CuH exhibiting the widest hysteresis.^42,43^

BET surface area, pore volume, and pore diameter of the five materials (Table 4) showed that 5CuH exhibited the highest specific surface area and pore volume among all samples. In contrast, 5CuH/Bent-3 had the lowest BET surface area, despite containing the same total mass of metal nitrates as 5CuH, but with an additional 3.000 g of bentonite.

**Table 4:** BET surface area, pore diameter, and pore volume of the synthesized materials

Sample	BET surface area (m^2^ g^−1^)	Pore diameter (nm)	Pore volume (cm^3^ g^−1^)
Bent	32.1	6.54	0.035
5CuH	72.7	8.33	0.140
5CuH/Bent-1	27.4	19.12	0.111
5CuH/Bent-2	31.1	16.01	0.107
5CuH/Bent-3	15.8	11.31	0.038

The BET surface areas of the three 5CuH/bentonite composites decreased significantly compared to 5CuH. However, 5CuH/Bent-1 and 5CuH/Bent-2 showed only minor reductions in BET surface area (4.7 and 1.0 m^2^ g^−1^, respectively) relative to pristine bentonite. Meanwhile, the pore volume and pore diameter of these two composites increased markedly compared to bentonite. In the case of 5CuH/Bent-3, the BET surface area dropped to 15.8 m^2^ g^−1^, the lowest among all five materials.

Overall, although the composites were less porous than 5CuH, they possessed well-developed mesopores with larger pore diameters and volumes than bentonite, which reflected changes in layer ordering and intercalation between bentonite and hydrotalcite, consistent with the XRD patterns and SEM observations discussed above.

In addition to factors such as surface functional groups, the BET surface area, pore volume, and pore diameter were critical parameters that influenced the adsorption performance of the synthesized materials. Several studies reported composites that exhibited significantly higher BET surface areas compared to the original bentonite, often attributed to the use or absence of ultrasonic treatment. For example, the BET surface areas of raw bentonite, acid-activated bentonite, and bentonite–hydrotalcite composites were reported as 27.84, 132.4, and 83.24 m^2^ g^−1^, respectively.^44^ Similarly, CoAl and bentonite-CoAl samples showed 44.20 and 119.14 m^2^ g^−1^.^31^ Bentonite, bentonite/polyaniline, and bentonite/polyaniline@Ni_2_O_3_ composites had 91, 127, and 231 m^2^ g^−1^;^45^ bentonite and bentonite-CuAl composites exhibited 38.87 and 82.81 m^2^ g^−1^;^30^ and B-Na^+^, Zn/Al-bent, and Mg/Al-bent showed 110.43, 175.95, and 209.25 m^2^ g^−1^.^35^ Typically, Na-bentonite displayed BET surface areas in the range of 20–30 m^2^ g^−1^, which could increase to 60–120 m^2^ g^−1^ upon Ca^2+^ modification^46^

Conversely, some composites showed a substantial decrease in BET surface area relative to raw bentonite. For instance, raw bentonite, ZnFe_2_O_4_, and ZnFe_2_O_4_/bentonite composite exhibited BET values of 56.3, 16.1, and 26.2 m^2^ g^−1^, respectively^47^ and raw bentonite and citric-acid-modified bentonite had similar BET values of 15.18 and 15.17 m^2^ g^−1^.^43^ In the present study, the composites synthesized from bentonite and 5CuH exhibited intercalation between bentonite layers and hydrotalcite sheets, resulting in reduced BET surface areas compared to the original bentonite and 5CuH samples.

#### 3.1.5. UV-Vis diffuse reflectance spectra of the materials (UV-Vis DRS)

**Fig. 7:**
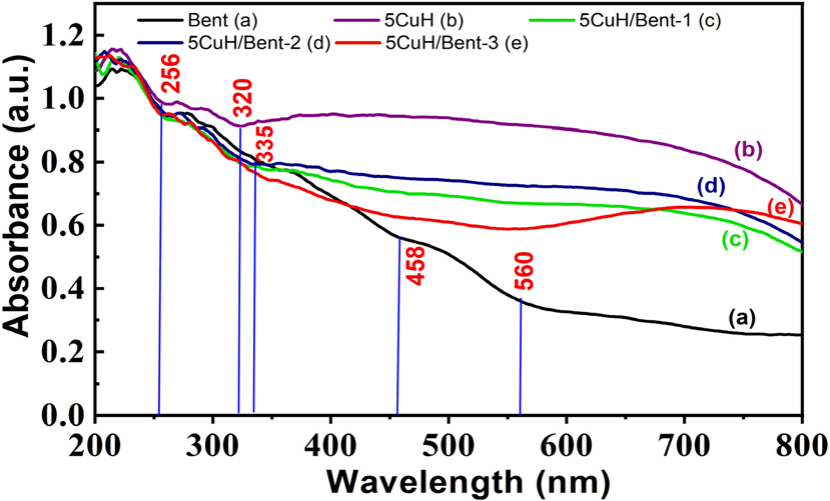
UV-Vis diffuse reflectance spectra (DRS) of the synthesized materials: Bent (a), 5CuH (b), 5CuH/Bent-1 (c), 5CuH/Bent-2 (d), and 5CuH/Bent-3 (e).

The UV-Vis DRS profiles of the five materials (Fig. 7) revealed three distinct absorption edges spanning the UV to visible regions. The first edge exhibited a pronounced absorption peak at approximately 220 nm, extending to about 256 nm. The second absorption edge appeared within 256–320 nm for the 5CuH sample and 256–335 nm for the 5CuH/Bent-1 and 5CuH/Bent-2 composites, which were attributed to charge transfer from O^2−^ to framework metal cations (Mg^2+^, Al^3+^, Si^4+^) in tetrahedral coordination. By contrast, the second absorption edge of the Bent and 5CuH/Bent-3 samples extended more broadly, from 256–458 nm and 256–560 nm, respectively. These features were assigned to charge transfer transitions from O^2−^ to Fe^3+^ and Ti^4+^ ions in bentonite, and from O^2−^ to Cu^2+^ in the 5CuH/Bent-3 composite.

The third absorption edge of 5CuH, 5CuH/Bent-1, and 5CuH/Bent-2 spanned 320–800 nm and 335–800 nm, respectively. These transitions were associated with charge transfer from O^2−^ to Cu^2+^ ions as well as d–d transitions of Cu^2+^ in octahedral coordination.^9,29^ For 5CuH/Bent-3, the d–d transitions were assigned to the 560–800 nm region, whereas for bentonite they occurred between 458–560 nm, consistent with the presence of Fe^3+^ and Ti^4+^ as confirmed by EDS analysis. Importantly, the red-shifted absorption edges of all five materials into the visible region suggested that these materials possessed photocatalytic activity under visible-light irradiation.

The optical band gap energies of the five materials were estimated using the Kubelka–Munk function in combination with Tauc plots. The Kubelka–Munk relation is expressed as:

8 (*αhν*) = *A*(*hν* − *E*_g_)^*n*^

where *α* is the absorption coefficient, *A* is a proportionality constant (often referred to as the band-tail parameter), *hν* is the photon energy, and *n* equals 1/2 for direct allowed transitions and 2 for indirect allowed transitions in semiconductors. The optical band gap energy (*E*_g_) of the synthesized catalysts was obtained by extrapolating the linear region of the (*αhν*)^2^*versus hν* plot to the intercept at *α* = 0. Since the energy required to excite an electron from the valence band (VB) to the conduction band (CB) depends on the band gap, photocatalysts with optimized *E*_g_ values in the visible-light region are expected to exhibit superior performance.^48^

Alternatively, the band gap can also be estimated from the absorption edge of the material using the following relation:

9 *E*_g_ = *hν* = *hc*/*λ* = 1240/*λ*

where *λ* is the wavelength corresponding to the intersection point between the tangent of the absorption edge and the horizontal axis.

In this study, the band gap was determined using the Kubelka–Munk function adapted for direct-transition-type semiconductors,^34^ expressed as:

10 (*αhν*)^2^ = *A*(*hν* − *E*_g_)

**Fig. 8:**
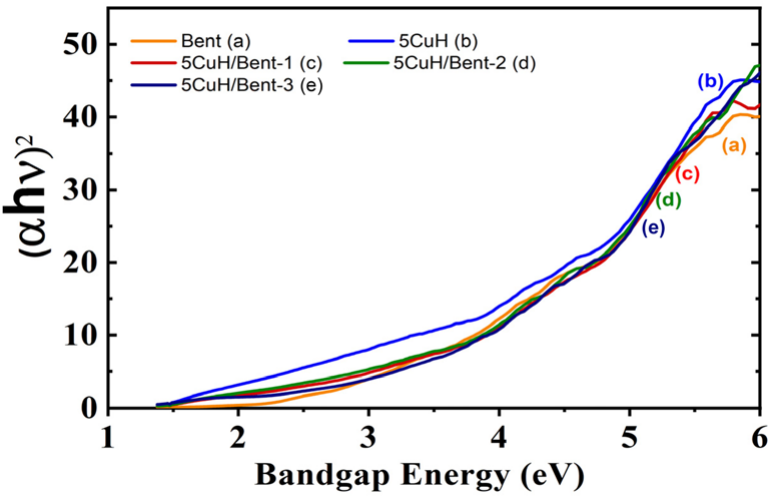
Tauc plots for determining the optical band gap energy (*E*_g_) of the synthesized materials: Bent (a), 5CuH (b), 5CuH/Bent-1 (c), 5CuH/Bent-2 (d), and 5CuH/Bent-3 (e).

The calculated band gap values of the five materials are presented in Fig. 8, S3 and Table S3 (SI).

As shown in Table S3 (SI), all five samples exhibited relatively narrow band gaps, ranging from 1.42 to 2.5 eV. In addition, the band gap values of 5CuH, 5CuH/Bent-1, 5CuH/Bent-2, 5CuH/Bent-3, and Bent corresponded to absorption edges in the UVA and UVB regions, consistent with the UV-Vis DRS results discussed above. The relatively low band gap of bentonite was attributed to the presence of Fe and Ti in its composition, which likely enhanced its photocatalytic response under visible light. Among the composite samples, the band gap values followed the order: 5CuH/Bent-3 > 5CuH/Bent-1 > 5CuH/Bent-2 > 5CuH. These band gap characteristics provided important insights into the potential photocatalytic activity of the synthesized materials for dye degradation.

### 3.2. Dark adsorption of RhB by the studied materials

**Fig. 9:**
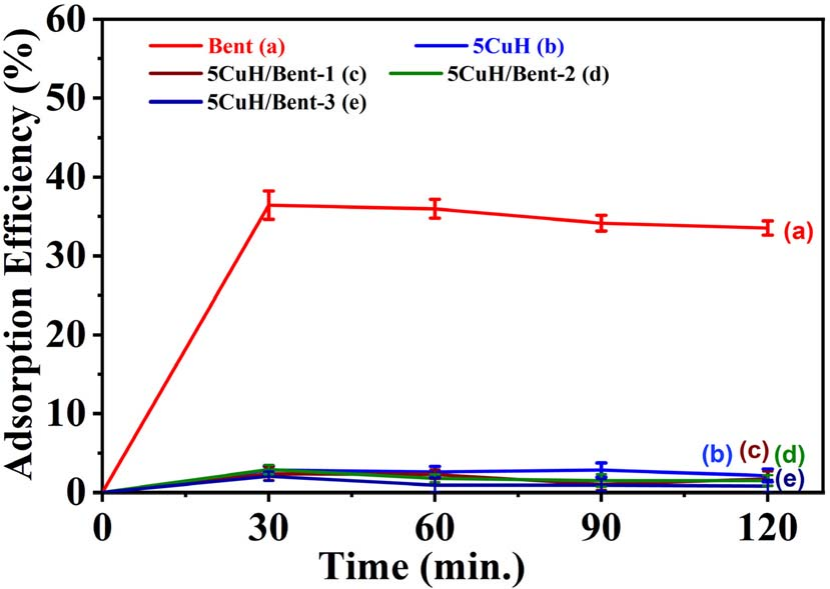
Adsorption efficiency of Rhodamine B (RhB, 50 mg L^−1^) as a function of contact time over the synthesized materials: Bent (a), 5CuH (b), 5CuH/Bent-1 (c), 5CuH/Bent-2 (d), and 5CuH/Bent-3 (e).

The adsorption behavior of 50 ppm Rhodamine B (initial pH = 5.20) on the five materials was investigated and the results are summarized in Table S4 (SI) and shown in Fig. 9. The adsorption efficiency increased during the first 30 minutes of dark stirring and remained nearly constant afterward. Therefore, an adsorption equilibrium time of 30 minutes was selected for all materials. The highest adsorption efficiency was observed for bentonite (36.4%), while the other four materials showed negligible RhB removal, with adsorption efficiencies ranging from 2.1 to 2.9%. Bentonite is an anionic clay and therefore exhibits strong affinity toward cationic species. Since Rhodamine B is a cationic dye, its adsorption efficiency on bentonite is the highest among the five tested materials. In contrast, the 5CuH hydrotalcite is a cationic layered clay, making it unfavorable for adsorbing the cationic Rhodamine B molecules.

For the three composite materials, 5CuH/Bent-1, 5CuH/Bent-2, and 5CuH/Bent-3, the higher proportion of 5CuH relative to bentonite likely results in interlayer intergrowth between hydrotalcite and bentonite, or even partial coverage of bentonite layers by hydrotalcite platelets. As a consequence, the adsorption behavior of these composites is dominated by the 5CuH component, which explains their uniformly low Rhodamine B adsorption efficiencies.

Rhodamine B is a cationic dye, whereas bentonite is a cation-exchange clay, and hydrotalcite is an anionic clay. Bentonite exhibited a strong cation-exchange capacity, allowing it to achieve 36.4% adsorption within 30 minutes.^35,44^ In contrast, the 5CuH hydrotalcite and the three composite materials exhibited minimal adsorption because they primarily facilitated anion adsorption and exchange, which was unfavorable for cationic RhB. This observation was consistent with previous reports.^49^

Furthermore, many hydrotalcite-based materials exhibited positive zeta potentials and high points of zero charge (pH_pzc_), resulting in positively charged surfaces that were unfavorable for cation adsorption. For instance, Mg–Al–CuFe–CO_3_ LDH had a pH_pzc_ of 10.45, while the calcined CLDH at 550 °C showed a pH_pzc_ of 11.73.^50^ The pH_pzc_ values of LDH, Cu_4_/LDH, and Cu_4_C/LDH were 9.9, 9.7, and 9.8, respectively^40^ and that of B-CuAl (bentonite-supported CuAl-LDH) was 6.3.^30^ For CoAl LDH and B-CoAl LDH, the pH_pzc_ values were 4.836 and 5.154, respectively.^31^ The apparent zeta potentials of MgAl-LDHs and CuO/MgAl-LDHs were +32.7 mV and +19.75 mV, respectively,^37^ and the 3CuAl sample exhibited a consistently positive zeta potential at pH ≤ 10.^29^ Therefore, under the initial solution conditions of 50 ppm RhB (pH 5.20), the hydrotalcite and 5CuH/Bent composite materials carried a net positive surface charge, which was unfavorable for RhB adsorption.

**Fig. 10:**
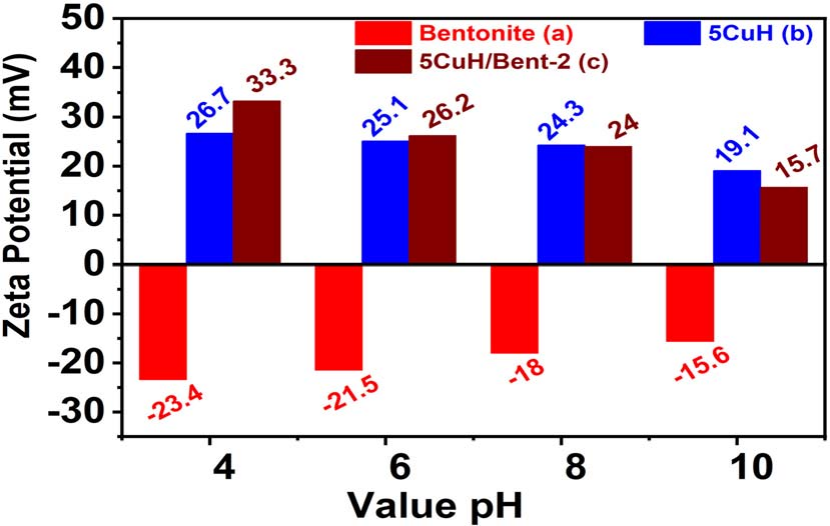
Apparent zeta potential of the three representative materials: Bent (a), 5CuH (b), and 5CuH/Bent-2 (c).

For the determination of apparent zeta potential, three representative materials, bentonite, 5CuH, and 5CuH/Bent-2, were selected. The corresponding data (Fig. 10) were obtained at pH 4.0, 6.0, 8.0, and 10.0. Bentonite carried negative surface charges throughout the investigated pH range. The measured values were −23.4, −21.5, −18.0, and −15.6 mV at pH 4.0, 6.0, 8.0, and 10.0, respectively, reflecting a gradual shift toward less negative values as the solution became more alkaline. In contrast, both 5CuH and 5CuH/Bent-2 maintained positive surface charges between pH 4.0 and 10.0. For 5CuH, the apparent zeta potential decreased markedly from 26.7 mV at pH 4.0 to 19.1 mV at pH 10.0. A similar tendency was recorded for 5CuH/Bent-2, with values declining from 33.3 mV to 15.7 mV over the same pH interval. This charge behavior aligns with the adsorption performance toward Rhodamine B discussed above. The negatively charged bentonite surface favors electrostatic attraction of the cationic dye, while the positive surface character of 5CuH-based materials explains the pH-dependent interaction patterns observed during the adsorption experiments.

The enhanced performance of the 5CuH/bentonite composite is closely associated with interfacial interactions between the negatively charged bentonite layers and the positively charged Cu-modified LDH platelets. Electrostatic attraction facilitates intimate interfacial contact and suppresses LDH aggregation, resulting in improved dispersion of catalytic domains, which has been shown to enhance interfacial charge transfer in heterostructured photocatalysts.

Clay supports not only increase surface area and pollutant enrichment but can also accelerate photogenerated electron transfer across the interface while inhibiting recombination, as reported for clay-based composites.

Meanwhile, layered double hydroxide-based composites have been widely observed to exhibit synergistic adsorption–photocatalysis mechanisms in pollutant removal, where adsorption enriches target molecules near active sites and enhances overall degradation efficiency.

### 3.3. Photocatalytic degradation of Rhodamine B under visible light irradiation

#### 3.3.1. Influence of irradiation time on photocatalytic performance

**Fig. 11:**
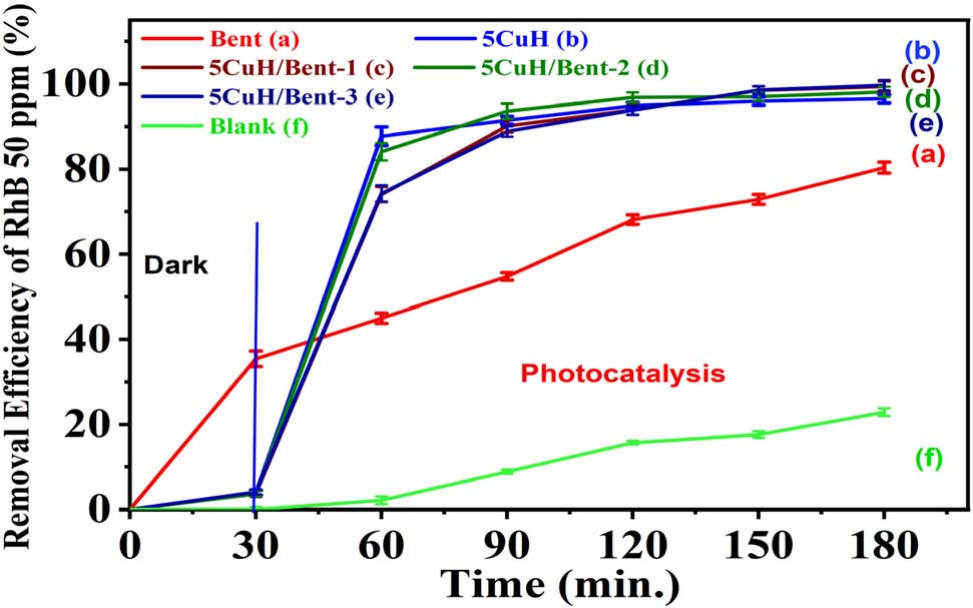
Removal efficiency of Rhodamine B (RhB, 50 mg L^−1^) over the synthesized materials under visible light irradiation using a 30 W LED lamp: Bent (a), 5CuH (b), 5CuH/Bent-1 (c), 5CuH/Bent-2 (d), 5CuH/Bent-3 (e), and blank (f).

The photocatalytic degradation of RhB (50 ppm) by the five synthesized materials was evaluated under visible light, and the results are summarized in Table S5 (SI) and Fig. 11.

As shown in Table S5 (SI) and Fig. 11, both 5CuH and the three composites (5CuH/Bent-1, 5CuH/Bent-2, and 5CuH/Bent-3) exhibited strong photocatalytic activity toward RhB degradation under visible light. After 180 min of irradiation, the degradation efficiencies of RhB (50 ppm) reached 92.7%, 95.7%, 94.4%, and 95.5% for 5CuH, 5CuH/Bent-1, 5CuH/Bent-2, and 5CuH/Bent-3, respectively. These results indicated that all four catalysts were able to achieve nearly complete degradation of RhB within 150 min. During the initial 60 min of irradiation, the photocatalytic activity of 5CuH and 5CuH/Bent-2 was higher than that of 5CuH/Bent-1 and 5CuH/Bent-3. Although the band gap energy of 5CuH was lower than that of the composites, its degradation efficiency was comparable to that of 5CuH/Bent-2 and higher than those of 5CuH/Bent-1 and 5CuH/Bent-3.

These findings suggested that combining bentonite with 5CuH at an appropriate ratio maintained high catalytic performance for RhB degradation while also lowering production costs, as bentonite is inexpensive and readily available. In particular, the amount of Cu, Mg, and Al nitrates required for the pure 5CuH material was about 1.5 times greater than that used in 5CuH/Bent-2, and the cost of these nitrates was substantially higher than that of bentonite.

When compared with pure bentonite, the four Cu-containing materials (5CuH, 5CuH/Bent-1, 5CuH/Bent-2, and 5CuH/Bent-3) showed markedly higher RhB removal efficiencies, highlighting the role of Cu^2+^ active centers in both the brucite-like layers and CuO crystallites in enhancing photocatalytic activity. Nevertheless, bentonite itself also exhibited relatively high activity, achieving 45% degradation of RhB (50 ppm) after 150 min and up to 80.4% after 180 min of irradiation. This performance was consistent with its band gap energy and the presence of Fe^3+^ and Ti^4+^ sites in the bentonite structure, which acted as active catalytic centers under visible light. In contrast, in the absence of a catalyst (H_2_O_2_ alone), RhB degradation reached only 22.8% after 150 min, which was in good agreement with a previous report,^51^ where 20.67% degradation was observed after 120 min of irradiation. These findings suggested that the synergistic combination of catalysts, H_2_O_2_, and visible light irradiation played a crucial role in improving photocatalytic performance. The underlying reactions responsible for this enhancement could be described as follows:^8,9,37,49^

11 5CuH/Bent + *hν* → 5CuH/Bent(e^−^, h^+^)

12 H_2_O_2_ + e^−^ → HO˙ + OH^−^

13 O_2_ + e^−^ → O_2_˙^−^

14 h^+^ + –OH (hydrotalcite) → HO˙

15 h^+^ + OH^−^ → HO˙

16 HO˙, O_2_˙^−^, h^+^ + RhB → intermediate oxidation products → … → final mineralization products (CO_2_ and H_2_O).

**Fig. 12:**
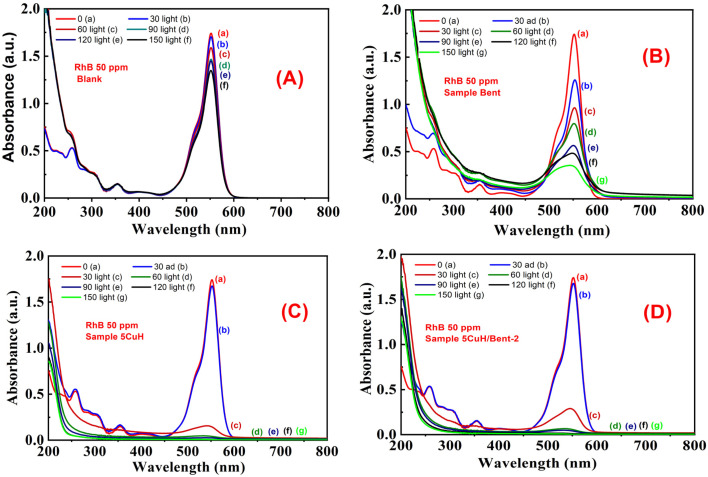
UV-Vis spectra of RhB (initial concentration 50 ppm) as a function of irradiation time under different conditions: (A) Blank, (B) Bent, (C) 5CuH, and (D) 5CuH/Bent-2.

The UV-Vis spectra of RhB solution (initial concentration 50 ppm) recorded over time under four conditions, (1) without catalyst (Blank sample, Fig. 12A), (2) with bentonite (Fig. 12B), (3) with 5CuH (Fig. 12C), and (4) with 5CuH/Bent-2 (Fig. 12D), revealed clear differences in degradation behavior. In the absence of a catalyst, the absorption peak of RhB at 553 nm decreased only slightly after 150 min of irradiation. With bentonite, the peak intensity decreased steadily, but a significant absorption band at 553 nm still remained after 150 min. By contrast, in the cases of 5CuH and 5CuH/Bent-2, the absorption peak at 553 nm dropped sharply within just 30 min of irradiation and almost disappeared completely after 60 min. Consequently, the RhB removal efficiencies of 5CuH and 5CuH/Bent-2 reached 91.5% and 93.6%, respectively, within only 60 min of visible-light exposure.

#### 3.3.2. Effect of RhB concentration on photocatalytic activity

**Fig. 13:**
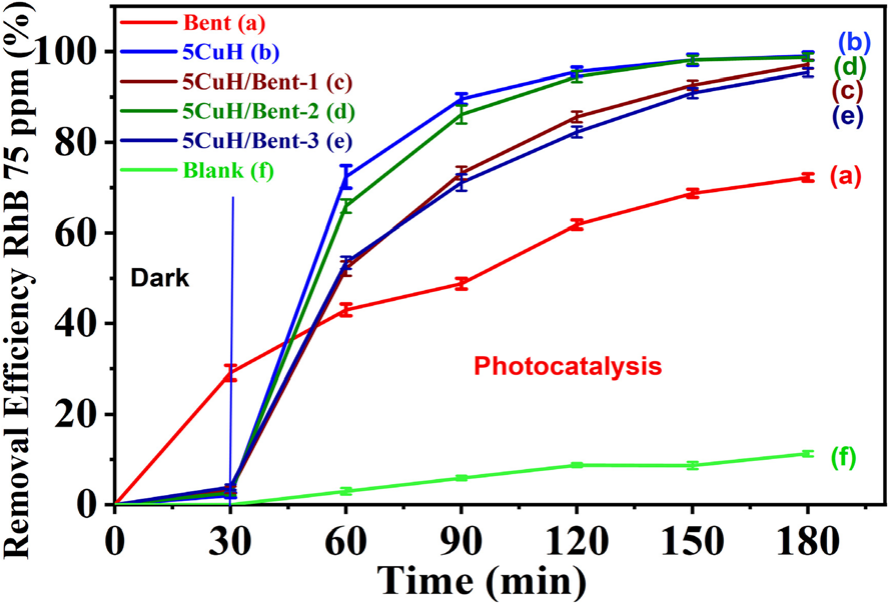
Removal efficiency of Rhodamine B (RhB, 75 mg L^−1^) by the synthesized composite materials under visible light irradiation: Bent (a), 5CuH (b), 5CuH/Bent-1 (c), 5CuH/Bent-2 (d), 5CuH/Bent-3 (e), and Blank (f).

**Fig. 14:**
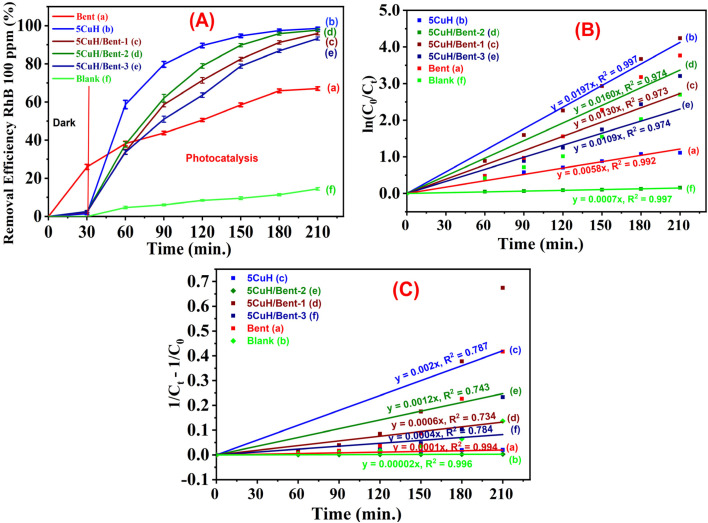
Removal efficiency of RhB (100 ppm) by the synthesized composite materials under visible light (A), and linear fitting of pseudo first order reaction kinetic plots (B)^52^ fitted curves of the pseudo-second-order kinetic model (C).^53^

To evaluate the ability of the five materials to degrade RhB at higher concentrations, RhB solutions of 75 ppm and 100 ppm were selected for investigation. The results are summarized in Tables S6, S7 (SI) and Fig. 13 and 14A.

From the obtained results, it can be observed that when the initial RhB concentration increased to 75 ppm and 100 ppm, the photocatalytic degradation rate was slower compared with that at the lower RhB concentration of 50 ppm (Fig. 13 and 14A). This outcome is consistent with the general trend that higher pollutant concentrations in solution result in slower degradation. Specifically, as the dye concentration increases, more dye molecules are adsorbed onto the material surface, which reduces the number of available active sites of the catalyst. At the same time, dye molecules can block or scatter the incident light, thereby reducing the generation of photoinduced electron–hole pairs, lowering the production of HO˙ radicals, and ultimately decreasing the photocatalytic degradation efficiency.^31,49,51^

**Fig. 15:**
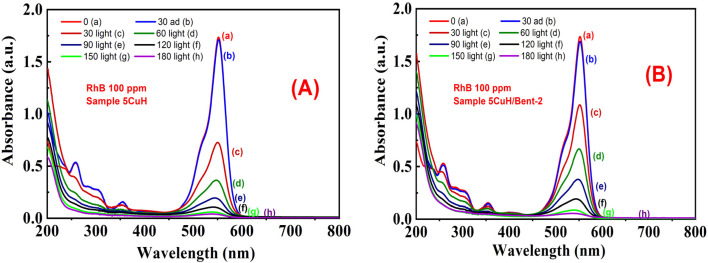
UV-Vis spectra of RhB recorded over time under irradiation in the presence of 5CuH (A) and 5CuH/Bent-2 (B) at an initial RhB concentration of 100 ppm.

When RhB removal was examined at these two higher concentrations, the degradation efficiency increased rapidly during the first 30 min of irradiation, followed by a gradual increase with prolonged irradiation. Accordingly, the UV-Vis spectra of RhB solution with an initial concentration of 100 ppm showed that the absorbance peak of RhB at 553 nm decreased sharply within the first 30 min of irradiation, and then gradually decreased at subsequent irradiation times of 60, 90, 120, and 150 min. After 180 min of irradiation, this peak almost completely disappeared for both 5CuH (Fig. 15A) and 5CuH/Bent-2 (Fig. 15B).

When examined at RhB concentrations of 75 and 100 ppm, the removal efficiencies of the four highly active materials, namely 5CuH, 5CuH/Bent-1, 5CuH/Bent-2, and 5CuH/Bent-3, reached 98.1, 92.6, 98.1, and 90.9% at 75 ppm after 180 min, and 98.6, 95.9, 97.7, and 93.3% at 100 ppm after 210 min of irradiation, respectively. In contrast, the removal efficiencies of RhB (75 and 100 ppm) by bentonite were significantly lower, only 68.7% and 67%, respectively. Moreover, in the absence of the catalyst (Blank sample), the degradation efficiencies of RhB (75 and 100 ppm) were negligible, with maximum values of only 14.8% and 14.5% after 180 and 210 min, respectively. Fig. S4 (SI) presents the evolution of the solution color of RhB with an initial concentration of 100 ppm during the reaction in the presence of three materials: bentonite (A, B), 5CuH (C), and 5CuH/Bent-2 (D). The test tubes contain RhB solution (100 ppm) together with intermediate degradation products formed during the reaction. For the 5CuH and 5CuH/Bent-2 systems (Fig. S4C and D), the initially deep red color gradually faded and became markedly lighter after 180 min of irradiation (corresponding to 210 min of total reaction time).

In contrast, when bentonite was used (Fig. S4A), the undiluted solutions retained a relatively strong coloration. The fading of the RhB solution and its intermediate products proceeded more slowly and remained less pronounced than in the two Cu-containing materials. A clearer visual difference appeared after ten-fold dilution of the solutions, as illustrated in Fig. S4B. Under these diluted conditions, the progressive decolorization becomes more distinguishable, reflecting the degradation of the 100 ppm RhB solution after 210 min of reaction in the bentonite system. In the kinetic analysis of RhB degradation, both first-order and second-order kinetic models can be applied. The mathematical expressions describing the first-order degradation kinetics of RhB (eqn (17)) and the second-order kinetics (eqn (18)) are given below:^53^

17 ln(*C*_0_/*C*_*t*_) = *k*_1_*t*

18 1/*C*_*t*_ − 1/*C*_0_ = *k*_2_*t*

Fig. 14B presents the pseudo-first-order kinetic profiles for the photocatalytic degradation of 100 ppm RhB by the synthesized materials. The reaction rate constants (*k*_1_) for 5CuH, 5CuH/Bent-2, 5CuH/Bent-1, 5CuH/Bent-3, and bentonite are 0.0197, 0.0160, 0.0130, 0.0109, and 0.0058 min^−1^, respectively. With regression coefficients (*R*^2^) ranging from 0.973 to 0.997, the pseudo-first-order model provides a good fit for the degradation kinetics of RhB at 100 ppm. The reaction rate constants of 5CuH, 5CuH/Bent-2, 5CuH/Bent-1, and 5CuH/Bent-3 are 3.4, 2.8, 2.2, and 1.9 times higher than that of bentonite, demonstrating that the incorporation of 5CuH catalytic centers in the three 5CuH/Bent composites significantly enhances their RhB degradation performance relative to pristine bentonite. Moreover, when compared with Cu_*x*_Ni_1−*x*_Fe_2_O_4_ (*x* = 0.4, 0.5) materials, which exhibit similar rate constants, both 5CuH and 5CuH/Bent-2 can effectively degrade RhB at much higher concentrations (100 ppm *vs.* 5, 7.5, and 15 ppm). This highlights the superior photocatalytic capability of the 5CuH and 5CuH/Bent-2 materials.

Fig. 14C presents the plots obtained from the second-order kinetic model for RhB degradation. The calculated rate constants for the degradation of 100 ppm RhB (*k*_2_, g mg^−1^ min^−1^) were 0.002 for 5CuH (*R*^2^ = 0.787), 0.0006 for 5CuH/Bent-1 (*R*^2^ = 0.734), 0.0012 for 5CuH/Bent-2 (*R*^2^ = 0.743), 0.0004 for 5CuH/Bent-3 (*R*^2^ = 0.784), and 0.0001 for Bent (*R*^2^ = 0.994). For all materials, these *k*_2_ values remain markedly lower than the corresponding *k*_1_ values derived from the first-order kinetic model. The correlation coefficients associated with the second-order model are also generally lower, with *R*^2^ ranging from 0.734 to 0.994. Under these conditions, the first-order kinetic model provides a more appropriate description of the RhB degradation process. Similar kinetic behavior has been reported previously by Aboraia *et al.* (2023).^53^

Furthermore, as shown in Fig. 13 and 14, there was a clear difference in photocatalytic activity among the four materials: 5CuH, 5CuH/Bent-1, 5CuH/Bent-2, and 5CuH/Bent-3. The order of photocatalytic activity decreased as follows: 5CuH > 5CuH/Bent-2 > 5CuH/Bent-1 > 5CuH/Bent-3. At lower RhB concentrations (50 and 75 ppm), the photocatalytic degradation efficiencies of 5CuH and 5CuH/Bent-2 were comparable, as were those of 5CuH/Bent-1 and 5CuH/Bent-3. However, at 100 ppm, the 5CuH sample exhibited superior photocatalytic activity compared with 5CuH/Bent-2 during the irradiation period of 30–60 min, with removal efficiencies approximately 17.7–21% higher. This result was attributed to the presence of CuO nanoparticles in the hydrotalcite framework, which enhanced the photocatalytic activity of the material.^37,40^ In addition, the greater number of CuO nanoparticles in 5CuH compared to 5CuH/Bent-2 (since the mass of metal nitrate precursors in 5CuH was 1.5 times higher than in 5CuH/Bent-2) further contributed to the superior photocatalytic activity of 5CuH during the first 120 min of irradiation.

In addition, the presence of CuO nanoparticles in these materials explains why the RhB removal efficiencies (75 and 100 ppm) of 5CuH and 5CuH/Bent-2 were much higher than that of CuH-3.0 (Cu : Zn : Al molar ratio = 3.0 : 4.0 : 3.0).^49^ Specifically, the RhB removal efficiencies at 100 ppm reached 94.7% and 89.8% after only 120 min of irradiation for 5CuH and 5CuH/Bent-2, respectively, whereas the CuH-3.0 sample required as long as 240 min of irradiation to achieve only about 90%. Fig. 15 shows the time-dependent UV-Vis spectra of RhB for the two samples, 5CuH (Fig. 15A) and 5CuH/Bent-2 (Fig. 15B), at an initial concentration of 100 ppm. The absorbance peak of RhB at 553 nm remained nearly unchanged after 30 min of adsorption–desorption equilibrium. However, the peak at 553 nm decreased sharply during the first 120 min of photocatalytic irradiation and completely disappeared after 180 min for both 5CuH and 5CuH/Bent-2. These results demonstrate that all four synthesized materials (5CuH, 5CuH/Bent-2, 5CuH/Bent-1, and 5CuH/Bent-3) are capable of efficiently removing RhB at high concentrations within relatively short irradiation times (150–180 min). In particular, 5CuH and 5CuH/Bent-2 exhibited significantly higher photocatalytic performance than 5CuH/Bent-1 and 5CuH/Bent-3.

**Fig. 16:**
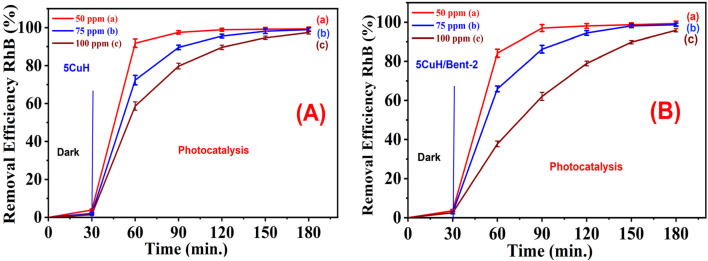
Comparison of RhB removal efficiency by 5CuH (A) and 5CuH/Bent-2 (B) at different initial RhB concentrations (50, 75, and 100 ppm).

Fig. 16A and B present a comparison of RhB degradation at initial concentrations of 50, 75, and 100 ppm for 5CuH and 5CuH/Bent-2, respectively. As the initial RhB concentration increased from 50 to 100 ppm, the photocatalytic degradation efficiency decreased. For both 5CuH and 5CuH/Bent-2, RhB degradation efficiencies above 90% were achieved within 90 min at concentrations of 50 and 75 ppm. However, at 100 ppm, a longer irradiation time of 120 min was required to reach approximately 90% degradation efficiency.

#### 3.3.3. RhB (75 ppm) removal in the dark by synthesized materials

Transition-metal-containing materials (Cu, Fe, Co, Ni, *etc.*) can act as heterogeneous Fenton-like catalysts when combined with oxidizing agents such as H_2_O_2_, peroxymonosulfate (PMS), or peroxydisulfate (PDS).^48,51^ In the review by Liu *et al.* (2021),^54^ hydroxyl radicals (HO˙) were described as highly reactive oxidants with a redox potential ranging from 1.89 to 2.8 V, while sulfate radicals (SO_4_˙^−^) possess comparable or even higher oxidative potential, ranging from 1.81 to 3.1 V. Among heterogeneous Fenton systems, Cu^2+^–H_2_O_2_ has been shown to generate reactive species more rapidly than Fe^2+^–H_2_O_2_. The formation of hydroxyl radicals (HO˙) from H_2_O_2_ in the presence of Cu^2+^-containing LDH materials occurs *via* the following reactions:^51^19
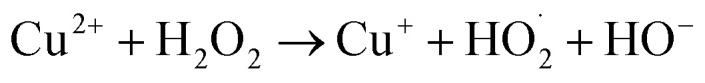

20 Cu^+^ + H_2_O_2_ → Cu^2+^ + HO˙ + OH^−^

Hydroxyl radicals (HO˙) can also be produced through the following reactions involving Co–Cu LDH materials.^55^

21 ≡Cu^2+^ + H_2_O_2_ → ≡Cu^3+^–OH + HO˙

22
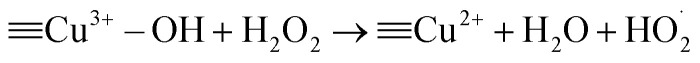

23 O_v_≡Cu^2+^ + H_2_O → O_v_≡Cu^2+^–H_2_O*

24
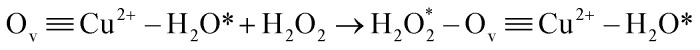
25
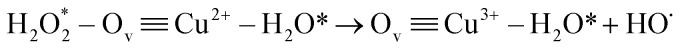
26
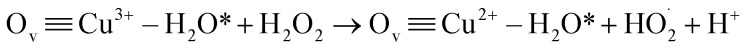
where, O_v_ is an oxygen vacancy.

27 After that, HO˙ + RhB → intermediate products → CO_2_ + H_2_O

Subsequently, the hydroxyl radicals participate in the degradation of RhB according to:

28 HO˙ + RhB → intermediated products → CO_2_ + H_2_O

**Fig. 17:**
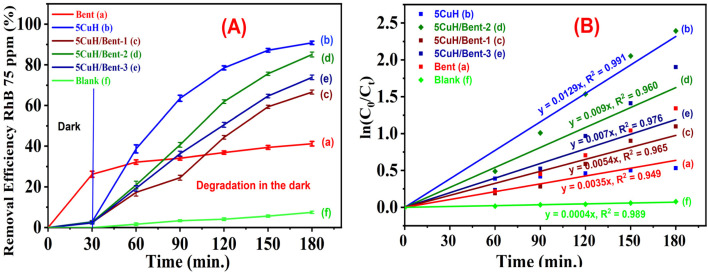
Removal efficiency of RhB (75 ppm) under dark conditions by the synthesized materials (A), and linear fitting of pseudo first order reaction kinetic plots (B).

The results of RhB degradation under dark conditions using the five synthesized materials are summarized in Table S8 (SI) and illustrated in Fig. 17A.

Based on the obtained results, all four synthesized materials, including 5CuH, 5CuH/Bent-1, 5CuH/Bent-2, and 5CuH/Bent-3, exhibited the ability to degrade RhB (75 ppm) in the absence of light (Fig. 17A). The degradation performance followed the order: 5CuH > 5CuH/Bent-2 > 5CuH/Bent-3 > 5CuH/Bent-1. Among them, the 5CuH sample achieved the highest RhB removal efficiency of 90.8%, while 5CuH/Bent-1 reached only 66.6% after 150 min under dark conditions. For comparison, bentonite exhibited a much lower efficiency of 41.2% under the same conditions, and in the absence of materials (with only H_2_O_2_ present), the removal efficiency was only 7.5% after 150 min.

These findings confirm that all four synthesized composites function as heterogeneous Fenton-like catalysts, reacting with H_2_O_2_ to generate hydroxyl radicals that drive the degradation of RhB. This catalytic activity can be attributed to the presence of Cu^2+^ centers in the brucite-like layers and CuO nanoparticles, both of which participate in the redox reactions (eqn (19) and (20)) described above to produce HO˙ radicals.

Fig. 17B shows the linear plots corresponding to the first-order kinetic model for the degradation of 75 ppm RhB in the dark. The reaction rate constants for the heterogeneous Fenton-like degradation of RhB by 5CuH, 5CuH/Bent-1, 5CuH/Bent-2, and 5CuH/Bent-3 are 0.0129 (*R*^2^ = 0.991), 0.0054 (*R*^2^ = 0.965), 0.009 (*R*^2^ = 0.960), and 0.007 min^−1^ (*R*^2^ = 0.976), respectively. These results confirm that first-order kinetics also adequately describe the RhB degradation process under dark conditions.

When compared with the first-order rate constant of Co–Cu LDH for the degradation of 10 ppm RhB in the dark (*k* = 0.2070 min^−1^), the reaction rate constants of 5CuH and 5CuH/Bent-2 for 75 ppm RhB are lower by factors of 16 and 23, respectively.^41^ In this study, the lower rate constants relative to Co–Cu LDH materials are attributed to the much higher RhB concentration used (75 ppm), which inherently slows the reaction kinetics.

**Fig. 18:**
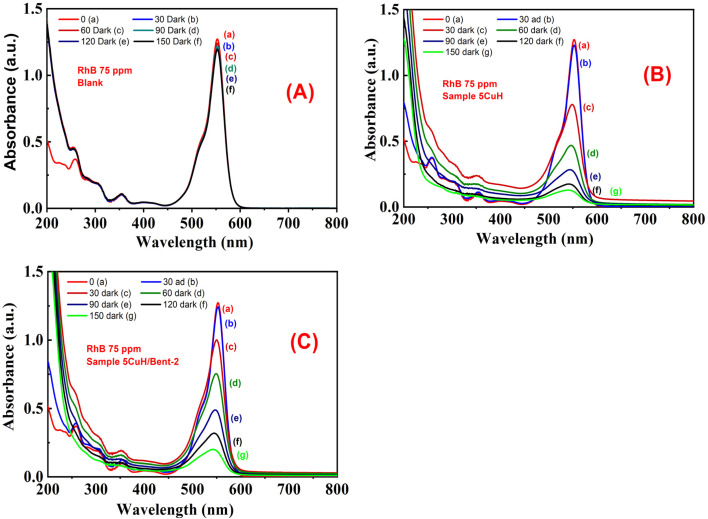
UV-Vis spectra of RhB (75 ppm) under dark conditions for Blank (A), 5CuH (B), and 5CuH/Bent-2 (C).

Fig. 18 presents the UV-Vis spectra illustrating the temporal changes in the absorption intensity of RhB. In the absence of a catalyst (Blank, only H_2_O_2_), the absorption peak at 553 nm of RhB decreased only slightly over time (Fig. 18A). By contrast, for the 5CuH/Bent-2 sample, the peak height gradually declined at a faster rate (Fig. 18C). It is worth noting that the 5CuH sample exhibited the most pronounced degradation effect, where the 553 nm absorption peak decreased more rapidly than in the case of 5CuH/Bent-2 (Fig. 18B). After 180 min of treatment, the RhB solution had become visibly lighter in the two beakers containing 5CuH and 5CuH/Bent-2.

**Fig. 19:**
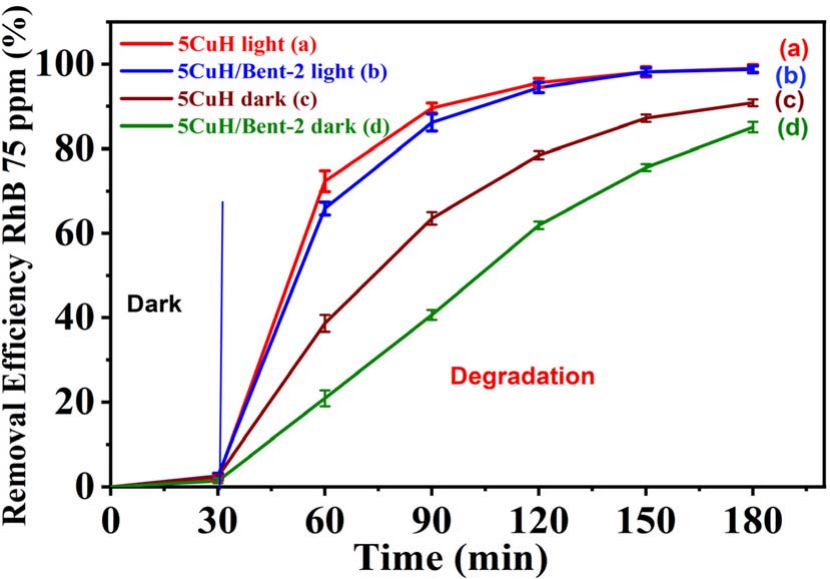
Comparison of Rhodamine B (RhB, 75 mg L^−1^) removal efficiency under different conditions: 5CuH under light irradiation (a), 5CuH/Bent-2 under light irradiation (b), 5CuH under dark conditions (c), and 5CuH/Bent-2 under dark conditions (d).

A comparison of the RhB (75 ppm) removal efficiency of 5CuH and 5CuH/Bent-2 under two conditions, with and without light irradiation, is presented in Fig. 19. It is evident that catalytic performance under light irradiation was significantly superior to that under dark conditions. After 60 min of irradiation, RhB removal efficiencies of 89.6% and 86.2% were achieved with 5CuH and 5CuH/Bent-2, respectively. In contrast, under dark conditions with H_2_O_2_, the efficiencies were only 63.5% and 40.7% for 5CuH and 5CuH/Bent-2, respectively. These results clearly demonstrate that light irradiation substantially enhances the catalytic activity of the synthesized materials, consistent with the findings reported by Zhang *et al.* (2020).^51^ The comparative results of RhB degradation by different catalytic systems in advanced oxidation processes are summarized in Table 5.

**Table 5:** Comparison of RhB removal efficiencies by various catalytic materials in different AOP systems

No	Sample	Reaction conditions	RhB removal efficiency	Ref.
1	5CuH	1.2 mL H_2_O_2_ 30% (0.047 mol L^−1^), 0.2 g catalyst, 250 mL RhB (50, 75, 100 ppm), 30 W LED	• 91.5% after 60 min irradiation (50 ppm RhB)	This study
			• 95.6% after 90 min irradiation (75 ppm RhB)	
			• 94.7% after 120 min irradiation (100 ppm RhB)	
	5CuH/Bent-2	1.2 mL H_2_O_2_ 30% (0.047 mol L^−1^), 0.2 g catalyst, 250 mL RhB (50, 75, 100 ppm), 30 W LED	• 93.6% after 60 min irradiation (50 ppm RhB)	
			• 94.5% after 90 min irradiation (75 ppm RhB)	
			• 89.8% after 120 min irradiation (100 ppm RhB)	
2	ZnFe_2_O_4_/bentonite (10%)	0.1 M H_2_O_2_, catalyst dosage 1 g L^−1^, 30 W LED	99.73% after 210 min irradiation	43
3	Cu_0.5_Ni_0.5_Fe_2_O_4_	0.05 M H_2_O_2_, catalyst dosage 1 g L^−1^, 100 mL RhB 7.5 ppm, 300 W vis lamp	97.25% after 120 min irradiation	56
4	Nd^3+^-doped CoFe_2_O_4_ (CoNd_0.05_Fe_1.95_O_4_)	0.1 M H_2_O_2_, 100 mL RhB 10 ppm, 30 W LED	94.7% after 180 min irradiation	57
5	Co–Cu_*t*=65°C_	10 mg Catalyst, 100 mL RhB 10 ppm, H_2_O_2_ 240 ppm, dark conditions	99.3% within 30 min	41
6	CuH-2.5, CuH-3.0, CuH-3.5	250 mL RhB 30 ppm, 0.2 g catalyst, 0.047 M H_2_O_2_, 30 W LED	90–93% after 30 min irradiation	49
		250 mL RhB (75, 100 ppm), 0.2 g catalyst, 0.047 M H_2_O_2_, 30 W LED	∼90% after 240 min irradiation	
7	Zn_2_SnO_4_/SnO_2_	50 mg Catalyst, 50 mL RhB 10 ppm, natural sunlight	70.6% after 120 min irradiation	58
8	CoFe_2_O_4_/g-C_3_N_4_	100 mL RhB 20 mg L^−1^, Osram 160 W lamp	72.28% after 60 min irradiation	59
	Nickel ferrite/(N,S)graphene oxide, NF/(N,S)GO	100 mL RhB (60, 80, 100 mg L^−1^), Osram 160 W lamp	87.5% (60 ppm RhB), 88.6% (80 ppm RhB), 95.2% (100 ppm RhB) after 240 min irradiation	
9	Polyaniline-coated XTiO_3_ (X = Co, Ni) nanocomposites, such as PANI@CoTiO_3_, PANI@NiTiO_3_	50 mL RhB 5 mg L^−1^, 50 W LED lamp, 7 W UV lamp	• 94% after 180 min irradiation with 50 W LED (PANI@NiTiO_3_)	60
			• 87% after 180 min irradiation with 7 W UV (PANI@NiTiO_3_)	

Table 5 compares several recently reported studies on RhB degradation, representing different approaches for treating this dye. From this comparison, it was evident that both 5CuH and 5CuH/Bent-2 showed high photocatalytic activity, as their removal efficiencies exceeded 90% for RhB concentrations of 50, 75, and 100 ppm within 60–120 minutes under 30 W LED irradiation. Most previous studies focused on much lower RhB concentrations (typically 10 ppm), whereas this work demonstrated that efficient degradation was still achieved at higher concentrations. This distinction highlighted one of the novel aspects of the study.

The two materials tested are superior to nickel ferrite/(N,S)-GO under conditions tested for RhB degradation (60, 80 and 100 ppm RhB) with both materials showing faster degradation rates and being more energy efficient than Osram Bulbs (160 W) at the same light intensity (30 W LED light) and presenting an additional novel contribution to the feasibility of energy-efficient visible light photocatalysis at such low power inputs.^59^ Furthermore, by using LED light, improved control of the photocatalytic process was achieved and thus the efficiency of the degradation of RhB was improved as this eliminated the deviations in conditions that can occur when using sunlight.^58^

A further benefit of these materials was the straightforward preparation of 5CuH and 5CuH/Bent-2 through a co-precipitation process at room temperature, with significantly lower costs due to the incorporation of Bent-5CuH and good photocatalytic efficiency. Overall, findings indicate that the use of these two materials provides increased performance over previously reported photocatalysts due to improved scalability, reduced manufacturing costs, improved energy efficiency (through use of greater numbers), and improved treatment capabilities for higher concentrations of dye loaded into solution. This work thus represents one of the very few reported instances of the use of a hydrotalcite-based catalyst with bentonite as the support material, yielding >90% removal of RhB (from 100 ppm) by low-power LED irradiation, establishing both scientific and practical bases for future industrial application related to wastewater treatment.

#### 3.3.4. Effect of solution pH on RhB removal efficiency

**Fig. 20:**
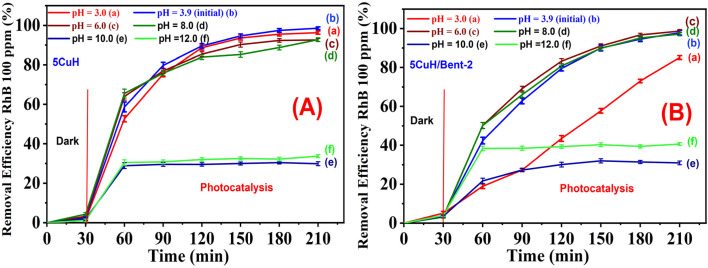
Removal efficiency of 100 ppm RhB under different pH conditions for 5CuH (A) and 5CuH/Bent-2 (B).

Two representative materials, 5CuH and 5CuH/Bent-2, were employed to investigate the influence of solution pH on the removal of RhB (100 ppm). The initial pH of the RhB solution was 3.9, and it was subsequently adjusted to 3.0, 6.0, 8.0, 10.0, and 12.0 for the experiments. The removal performances of the two materials under these different pH conditions were summarized in Tables S9 and S10 (SI), while the overall trends were illustrated in Fig. 20.

Both 5CuH and 5CuH/Bent-2 show a significant drop in their ability to absorb or destroy light under strongly basic conditions (pH between 10.0 and 12.0); however, the absorption of RhB (100 ppm) by either of these materials showed no significant variation within the full pH range (between 3.0–12.0). Under strongly acidic conditions (3.0), the materials exhibited a noticeable difference in their photocatalytic activity; at this pH, 5CuH achieved 93.6% removal of RhB after 150 minutes, whereas the composite material 5CuH/Bent-2 achieved only 57.7% removal of RhB. The difference was attributed to partial dissolution of CuO and Cu(OH)_2_ in the brucite lattice of 5CuH, resulting in the establishment of a homogeneous Cu^2+^–H_2_O_2_ Fenton-like catalytic system, effectively increasing its catalytic activity.^51^ These observations were consistent with previous studies on Phenol Red removal using 5CuH and 5CuH500 at pH 3.0,^9^ as well as ciprofloxacin removal using CuH and CuCoH_4_ at pH 3.0.^29^

Conversely, 5CuH/Bent-2 exhibited lower photocatalytic activity under acidic conditions (pH 3.0), likely due to stronger binding of CuO and Cu^2+^ within the brucite layers, caused by the intercalation of hydrotalcite and bentonite layers. This structural feature reduced the amount of Cu^2+^ ions released into the solution, resulting in lower catalytic performance compared to 5CuH.

However, the partial dissolution of CuO and Cu(OH)_2_ in the brucite lattice at pH 3.0 significantly reduced the photocatalytic activity of 5CuH/Bent-2 compared to its performance at the initial pH, pH 6.0, and pH 8.0. In general, when LDH or hydrotalcite materials undergo dissolution, their structure is altered and the number of active catalytic sites decreases, resulting in a decline in photocatalytic activity.^41,49^ The observed optimal pH range for 5CuH/Bent-2 was consistent with the reported optimal pH range for bentonite/polyaniline@Ni_2_O_3_ (BE/PANI@Ni_2_O_3_) composites.^45^

**Fig. 21:**
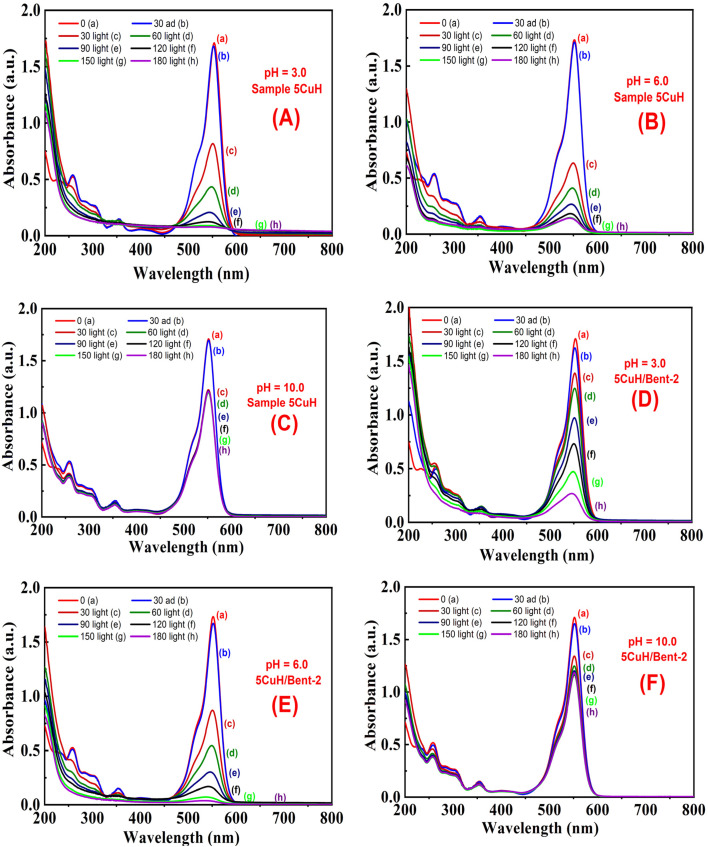
Presents the UV-Vis absorption spectra of Rhodamine B (RhB) as a function of irradiation time under different pH conditions. (A–C) 5CuH at pH 3.0 (A), 6.0 (B), and 10.0 (C); (D–F) 5CuH/Bent-2 at pH 3.0 (D), 6.0 (E), and 10.0 (F).

Upon illumination of 5CuH, the peak intensity at 553 nm decreased sharply over time at pH 3.0 and 6.0, while it remained almost unchanged at pH 10.0 (Fig. 21A–C). Significantly, the peak at 553 nm declined more rapidly at pH 6.0 than at pH 3.0, indicating that a mildly acidic environment (pH 6.0) promoted higher degradation efficiency compared to a strongly acidic environment (pH 3.0). In contrast, under strongly basic conditions (pH 10.0), RhB underwent negligible degradation.

A similar trend was observed for 5CuH/Bent-2 at pH 6.0 and 10.0 (Fig. 21E and F). At pH 6.0, the 553 nm peak decreased rapidly upon illumination and RhB was completely degraded after 180 minutes. Conversely, the peak intensity at 553 nm decreased slowly at pH 3.0 (Fig. 21D) and remained relatively high, with the solution retaining a pink color even after 180 minutes of illumination.

In addition to these factors the influence of reaction temperature on degradation behavior should also be considered. All photocatalytic experiments were performed at ambient temperature (25 ± 2 °C). Under visible-light irradiation, the reaction temperature remained stable due to continuous stirring and effective heat dissipation. In this system, dark adsorption contributed only marginally to RhB removal (≈2–3%), indicating that degradation is primarily driven by photocatalytic oxidation rather than adsorption equilibrium. While temperature variations can influence adsorption–desorption balance and surface reaction kinetics in adsorption-dominated systems,^61^ the present process is governed mainly by photoinduced charge carrier generation and reactive oxygen species formation. Therefore, within the investigated temperature range, the degradation efficiency is controlled predominantly by photochemical rather than thermal effects. Further temperature-dependent kinetic studies may provide additional insight into activation energy and surface reaction dynamics and will be explored in future investigations.

#### 3.3.5. Proposed mechanism for RhB degradation by the materials

**Fig. 22:**
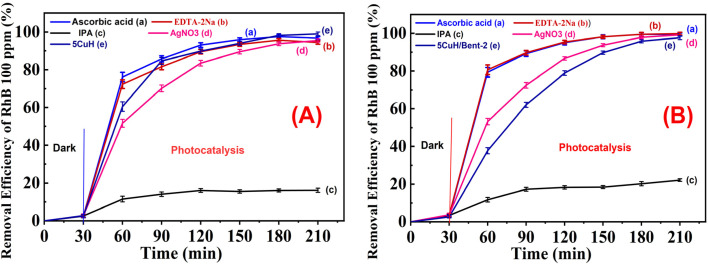
Removal efficiency of 100 ppm RhB at the initial pH (pH = 3.9) for 5CuH (A) and 5CuH/Bent-2 (B) in the presence of electron (e^−^), hole (h^+^), and reactive oxygen species scavengers.

To identify the reactive species and photogenerated electron–hole pairs directly involved in the photocatalytic degradation of RhB, scavenger experiments were conducted. In these experiments, isopropyl alcohol (IPA), ascorbic acid, AgNO_3_, and disodium ethylenediaminetetraacetate (EDTA-2Na) were employed to quench hydroxyl radicals (˙OH), superoxide radicals (O_2_˙^−^), photogenerated electrons, and photogenerated holes, respectively.^29,33,37^ The results of the scavenger tests for 5CuH and 5CuH/Bent-2 were summarized in Tables S11 and S12 (SI), and illustrated in Fig. 22.

From the results presented in Tables S11, S12 (in SI) and Fig. 22, it was concluded that photogenerated hydroxyl radicals (˙OH) directly participated in the oxidation of RhB, producing intermediate degradation products that were ultimately mineralized to CO_2_ and H_2_O. When 30% H_2_O_2_ was applied in the presence of the materials and the reaction mixture was illuminated, photogenerated electron–hole pairs (e^−^–h^+^) contributed to the transformation of H_2_O_2_ and the –OH groups in hydrotalcite and bentonite, generating reactive hydroxyl radicals. These radicals directly participated in the photocatalytic degradation of RhB in solution.

The photocatalytic degradation process produced multiple intermediate compounds. Rhodamine B, with *m*/*z* = 443 g mol^−1^, was transformed into intermediates with *m*/*z* values of 415, 387, 359, 331, 282, 226, 181, 167, 148, 132, 122, 116, 90, and others. These intermediates were further degraded into smaller molecules and eventually mineralized to CO_2_ and H_2_O.^59,60,62^ According to acute and chronic toxicity studied on green algae, fish, and Daphnia by Mohod *et al.* (2023), intermediate products with molecular weights between 232 and 443 g mol^−1^ were highly toxic, showing LD_50_, EC_50_, and ChV values all below 1.0. In contrast, intermediates with *m*/*z* values between 90 and 155 g mol^−1^ exhibited lower acute and chronic toxicity, with many compounds being non-toxic. In general, smaller molecular weight products displayed lower toxicity or were non-toxic.^62^

**Fig. 23:**
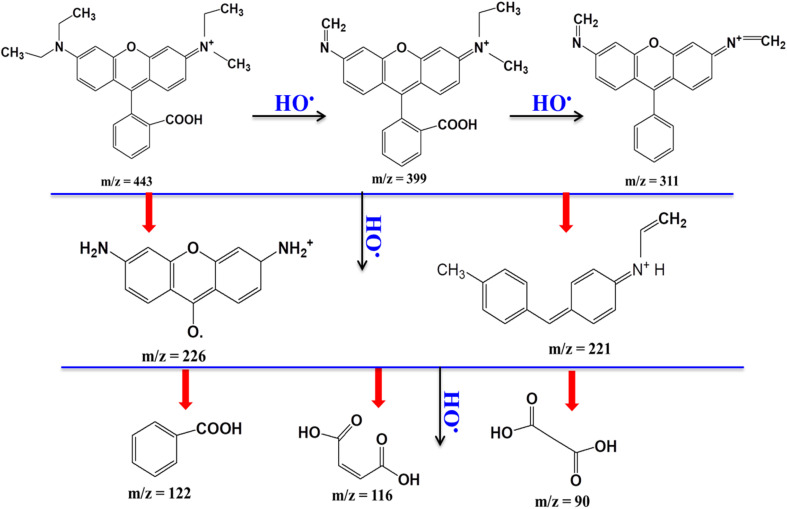
Chemical structures of selected intermediate products formed during the photocatalytic degradation of RhB.^59,60,62^

**Fig. 24:**
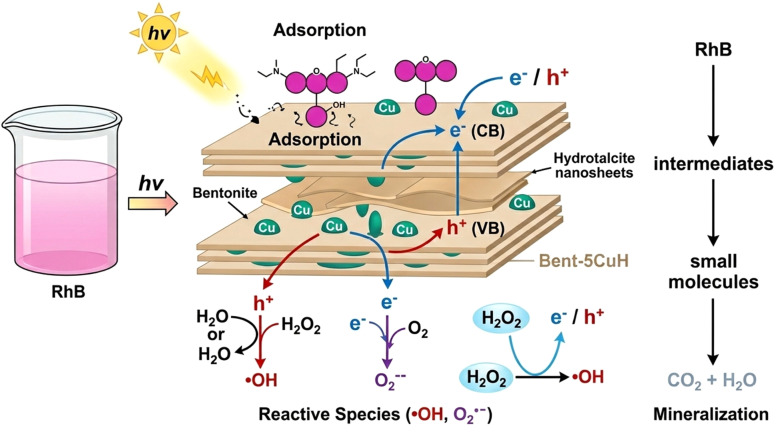
Proposed photocatalytic degradation mechanism of Rhodamine B (RhB) over the 5CuH/Bent composite under light irradiation.

Therefore, to ensure that RhB was degraded into non-toxic or low-toxicity products, it had to be rapidly degraded and mineralized to form smaller molecules with molecular weights below 155 g mol^−1^. Fig. 23 illustrates some of the intermediate products generated during RhB treatment reported in the previous studies.^59,60,62^ Based on the identified intermediate products and their progressive transformation into smaller and less toxic molecules, a plausible photocatalytic degradation mechanism of RhB over the 5CuH/Bent composite under light irradiation was proposed, as illustrated in Fig. 24.

Upon irradiation (*hν*), photogenerated electrons (e^−^) are excited to the conduction band (CB) of the Cu-modified Mg–Al hydrotalcite nanosheets, leaving holes (h^+^) in the valence band (VB). The layered bentonite support facilitates the dispersion of Cu species and enhances charge separation within the 5CuH/Bent structure. The photogenerated electrons react with dissolved O_2_ to form superoxide radicals (O_2_˙^−^), while the holes oxidize H_2_O or H_2_O_2_ to generate hydroxyl radicals (˙OH). Meanwhile, RhB molecules are first adsorbed on the catalyst surface through electrostatic interactions and surface functional groups. These reactive oxygen species (˙OH and O_2_˙^−^) subsequently attack the adsorbed RhB molecules, leading to stepwise degradation into intermediate compounds, followed by smaller organic molecules, and ultimately mineralization into CO_2_ and H_2_O.

### 3.4. Reusability and stability

**Fig. 25:**
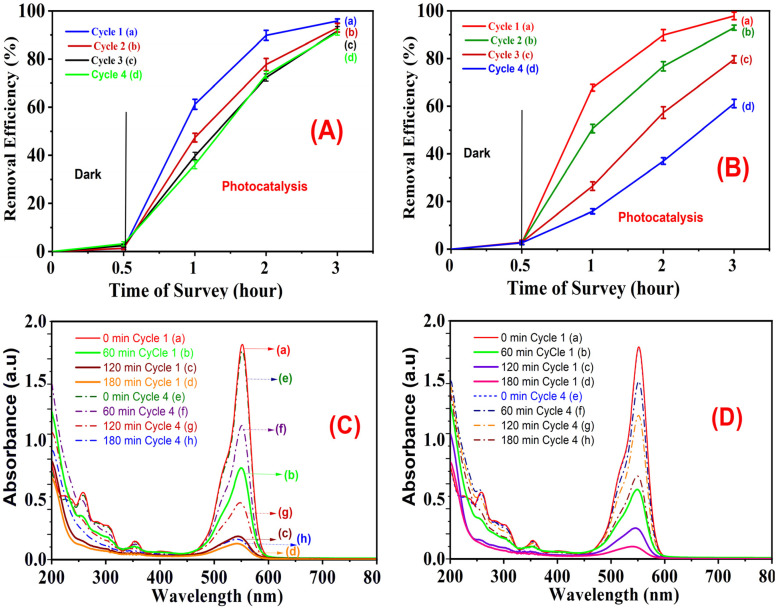
RhB (100 ppm) degradation efficiency over time during four consecutive reuse cycles of (A) 5CuH and (B) 5CuH/Bent-2, and UV-Vis absorption spectra of RhB solution after 180 minutes of irradiation during the first and fourth cycles for (C) 5CuH and (D) 5CuH/Bent-2.

The results of the stability and reusability tests for the two materials, 5CuH (Fig. 25A) and 5CuH/Bent-2 (Fig. 25B), showed that the photocatalytic activity of both materials decreased after each reuse cycle, with a more pronounced decline observed for 5CuH/Bent-2. For the 5CuH sample, the RhB (100 ppm) degradation efficiency slightly decreased during the first 120 minutes of irradiation and dropped by only 4.7% after 180 minutes following four reuse cycles. The degradation efficiencies of 5CuH after 180 minutes of illumination were 95.8%, 92.9%, 91.7%, and 91.1% for the first, second, third, and fourth runs, respectively.

In contrast, the photocatalytic activity of 5CuH/Bent-2 declined markedly at all illumination intervals (60, 120, and 180 minutes) during the four reuse cycles. After 180 minutes of irradiation, the RhB (100 ppm) removal efficiency decreased from 97.8% in the first run to 92.9%, 79.6%, and 61.1% in the second, third, and fourth runs, respectively, corresponding to a total decrease of 31.8% after four uses. These findings indicated that the 5CuH material retained its photocatalytic activity much better than the 5CuH/Bent-2 composite over repeated use.

The UV-Vis absorption spectra of RhB solution during illumination with the 5CuH photocatalyst (Fig. 25C) showed that the intensity of the main absorption peak at 553 nm in the fourth reuse cycle (dashed curves) was slightly higher than that observed in the first cycle (solid curves) at 60 and 120 minutes of irradiation. However, the difference between the two cycles became insignificant after 180 minutes. In contrast, for the 5CuH/Bent-2 sample (Fig. 25D), the absorption peak at 553 nm remained noticeably higher in the fourth cycle than in the first one at all corresponding illumination times, indicating a stronger decrease in photocatalytic performance.

**Fig. 26:**
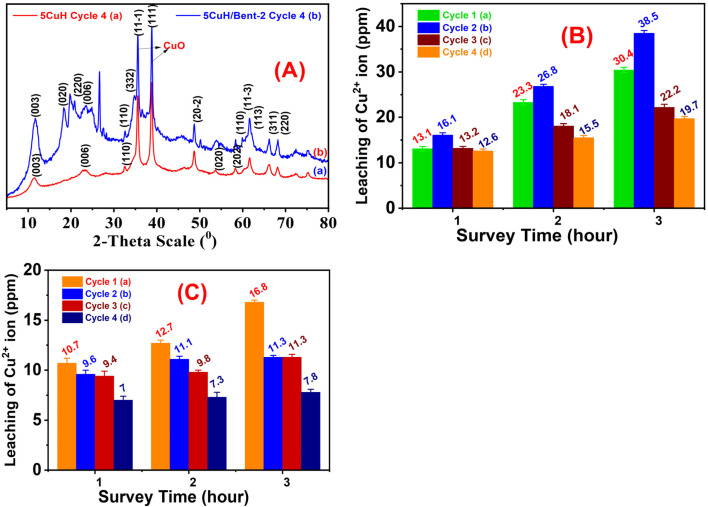
XRD patterns of 5CuH and 5CuH/Bent-2 after four reuse cycles (A), and the concentrations of dissolved Cu^2+^ ions in solution over time during the four consecutive reuse cycles of (B) 5CuH and (C) 5CuH/Bent-2.

Quantitative analysis of Cu^2+^ ions in the reaction solution revealed that the concentration of dissolved Cu^2+^ released from 5CuH was considerably greater than that from 5CuH/Bent-2. After 3 hours of illumination, the Cu^2+^ concentrations in solution reached 30.4, 38.5, 22.2, and 19.7 mg L^−1^ for the first to fourth cycles of 5CuH, respectively (Fig. 26B), corresponding to a cumulative Cu^2+^ loss of 110.8 mg L^−1^ after four uses. In comparison, the 5CuH/Bent-2 material released lower Cu^2+^ concentrations of 16.8, 11.3, 11.3, and 7.8 mg L^−1^ for the respective cycles (Fig. 26C), resulting in a total loss of 47.2 mg L^−1^.

The progressive dissolution of Cu^2+^ ions into the solution after each reuse cycle was identified as the main reason for the reduction in photocatalytic activity and the decline in RhB degradation efficiency. In the case of the 5CuH/Bent-2 composite, the incorporation of bentonite with 5CuH appeared to suppress Cu^2+^ leaching to some extent; however, the loss of Cu^2+^ active sites still caused a pronounced decrease in photocatalytic efficiency after repeated use. Interestingly, the XRD patterns of both 5CuH and 5CuH/Bent-2 after four reuse cycles (Fig. 26A) exhibited no significant differences compared with those of the fresh materials before RhB degradation. This finding suggested that both photocatalysts retained good structural stability, although the partial dissolution of Cu^2+^ ions adversely affected their catalytic performance, particularly in the 5CuH/Bent-2 sample.

### 3.5. Application in real textile wastewater treatment

#### 3.5.1. Removal efficiency of dyes (RhB and others if present)

**Fig. 27:**
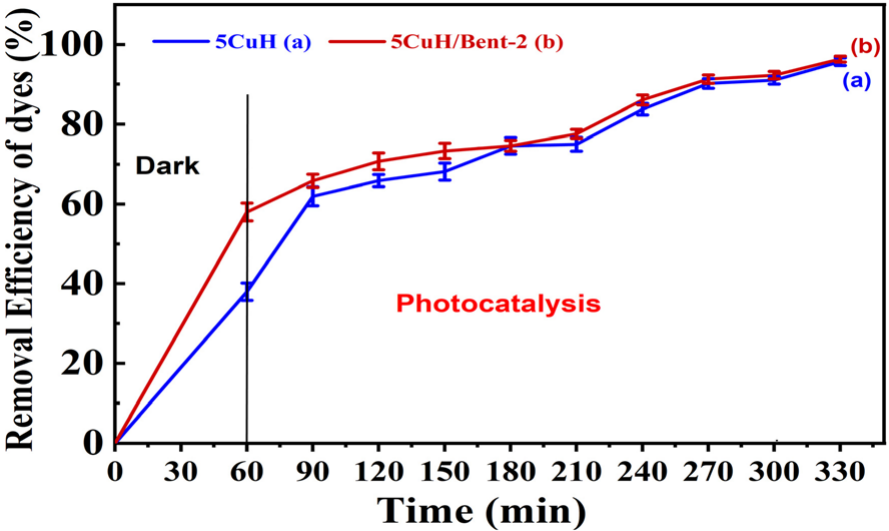
Removal efficiency of dyes in mat-dyeing textile wastewater by 5CuH (a) and 5CuH/Bent-2 (b).

To evaluate the practical applicability of the synthesized materials for wastewater treatment in the textile industry, two materials, 5CuH and 5CuH/Bent-2, were further employed to investigate their ability to degrade dyes and mineralize pollutants present in real textile wastewater from mat dyeing. The wastewater was collected from a small-scale craft workshop in Quynh Phụ commune, Thai Binh province. The untreated wastewater was dark red, highly colored. After a 15-fold dilution, the wastewater exhibited a pinkish-red color with a color value of 775 on the Pt–Co scale. The measured pH was 5.67, and the total suspended solids (TSS) concentration reached 75 mg L^−1^. The treatment procedures were conducted as described in the Experimental section 2.8 (see Materials and methods section). The results of this investigation are presented in Table S13 (SI) and Fig. 27.

Based on the absorbance values measured at 551–552 nm, the initial absorbance (Abs_0_) and the absorbance at each sampling time (Abs_*t*_) were determined. From these values, the dye removal efficiency of the two materials was calculated. The results presented in Table S13 (SI) and Fig. 27 showed that after 60 minutes of stirring in the dark, the adsorption efficiency of dyes on 5CuH and 5CuH/Bent-2 reached 37.9% and 58.2%, respectively.

This indicated that both materials exhibited good adsorption capacities for various dyes in real wastewater, with 5CuH/Bent-2 performing better than 5CuH. Notably, these results differed from the adsorption behavior observed for RhB alone. This difference was attributed to the complex composition of the textile wastewater, which contained multiple dye types, particularly those absorbing at 551–552 nm that were effectively adsorbed by both materials.

Upon illumination of the reaction mixture, the rate of dye degradation increased gradually over time. Unlike the case of single RhB treatment, no rapid increase was observed between 30 and 60 minutes of illumination. After 270 minutes, the dye removal efficiencies reached 95.6% and 96.3% for 5CuH and 5CuH/Bent-2, respectively. Remarkably, 5CuH/Bent-2 achieved higher removal efficiency than 5CuH, which was attributed to its superior adsorption performance, resulting in enhanced photocatalytic degradation of the dyes.^51^ The temporal evolution of the dye color during the treatment process is presented in Fig. S5A and B (see in SI). When the dye solutions were observed without dilution, pronounced color variations became visible throughout the reaction period. The initially deep red solution gradually faded and appeared much lighter after 300 min of reaction, corresponding to 4 h of irradiation.

**Fig. 28:**
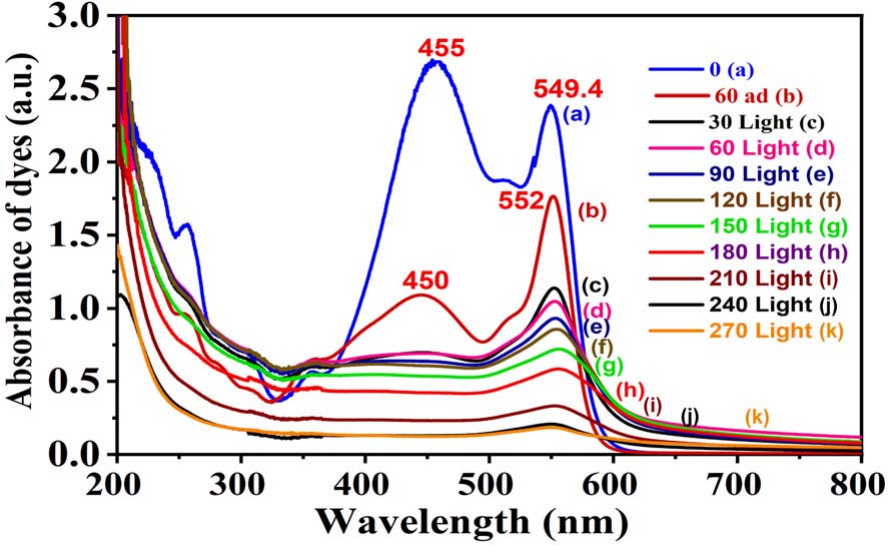
UV-Vis spectra of dyes in wastewater as a function of time over 5CuH/Bent-2: initial (0 min) (a), after adsorption in the dark for 60 min (b), and under light irradiation for 30 min (c), 60 min (d), 90 min (e), 120 min (f), 150 min (g), 180 min (h), 210 min (i), 240 min (j), and 270 min (k).

The UV-Vis spectra of the dye mixture in the wastewater over time, examined with 5CuH/Bent-2 (Fig. 28), showed that initially, before treatment, the wastewater exhibited two prominent absorption peaks at 551 nm and 455 nm. After 60 minutes of stirring in the dark to assess the adsorption capacity of the material, the spectra showed peaks at 552 nm and 450 nm, with both peak intensities significantly decreased compared to the initial values, particularly the peak at 450 nm, which exhibited the largest reduction.

During the illumination period from 30 to 270 minutes, the absorption peak at 450 nm nearly disappeared after 30 minutes of illumination, while the peak at 552 nm gradually decreased in intensity with increasing illumination time. After 270 minutes of illumination, the red color of the reaction mixture had noticeably faded compared to the initial mixture. The overall dye removal efficiency reached 96.3% after a total of 330 minutes of treatment.

#### 3.5.2. Mineralization capability of the materials for dyes

**Fig. 29:**
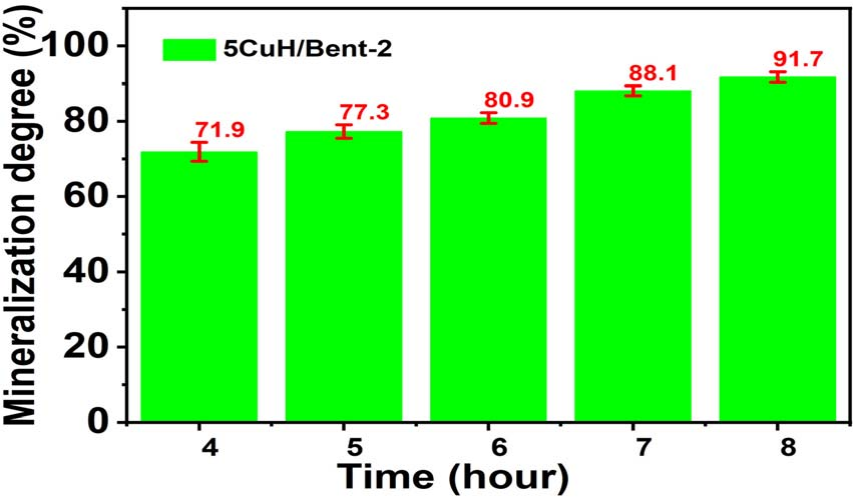
Mineralization of 100 ppm RhB by 5CuH/Bent-2 at the initial pH = 6.0.

To evaluate the mineralization capability of the synthesized 5CuH/Bent-2 material for RhB, after complete decolorization of the RhB solution, the reaction mixture was further illuminated to monitor the decrease in COD values. The results (Table 6 and Fig. 29) showed that the COD gradually decreased and the degree of dye mineralization increased with treatment time. This trend contrasted with the rate of color removal by the material. While the removal efficiency of 100 ppm RhB reached 97.7% after only 180 minutes of illumination, resulting in almost complete decolorization, the initial COD of the 100 ppm RhB solution (185.3 mgO_2_ per L) decreased to 15.3 mgO_2_ per L only after 8 hours of illumination. Consequently, an extended illumination time of 8 hours was required to achieve a mineralization efficiency of 91.7%. The investigation indicated that the mineralization rate was much slower than the decolorization rate. Therefore, to treat RhB without generating toxic intermediate products, it was necessary to prolong the reaction time to enhance the material's mineralization efficiency, even though the solution had already become completely colorless.

**Table 6:** Mineralization of 100 ppm RhB solution by 5CuH/Bent-2 as indicated by COD values at pH = 6.0

Time (hour)	COD (mgO_2_ per L)	Mineralization (%)
0	185.3	0.0
4	52.0	71.9
5	42.0	77.3
6	35.3	80.9
7	22.0	88.1
8	15.3	91.7

**Fig. 30:**
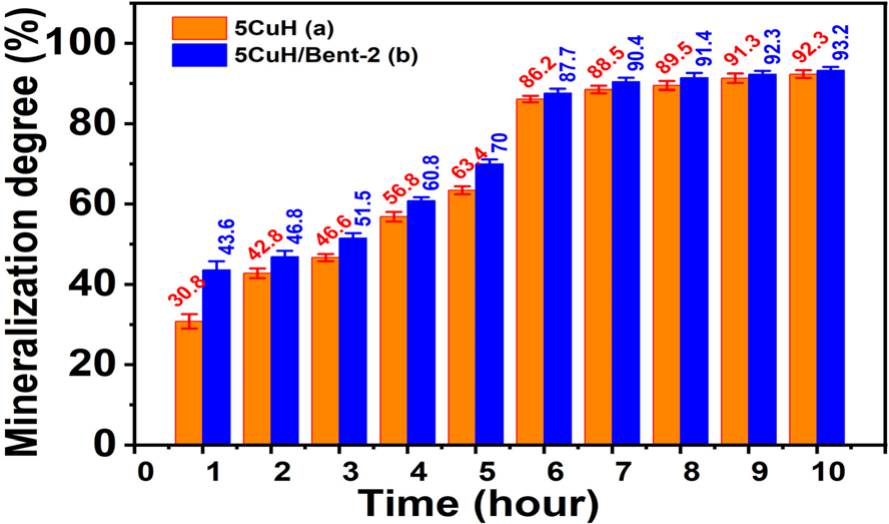
Mineralization of dyes in mat-dyeing textile wastewater by 5CuH (a) and 5CuH/Bent-2 (b).

In addition to assessing the mineralization of RhB, experiments were also conducted to monitor changes in the COD index over time during the treatment of textile wastewater and to evaluate the overall mineralization efficiency of the dyes by the synthesized materials. In this experiment, aliquots of the reaction mixtures containing 5CuH and 5CuH/Bent-2 were collected at one-hour intervals, centrifuged to remove residual solids, and subsequently analyzed for COD according to the procedure described in Section 2.9 (see Materials and methods section). The obtained results are summarized in Table S14 (SI) and illustrated in Fig. 30.

After the textile wastewater was diluted tenfold to prepare the samples for investigating dye removal and mineralization of pollutants, the initial COD values were 715.3 and 718.3 mgO_2_ per L for the 5CuH and 5CuH/Bent-2 samples, respectively. Following one hour of adsorption, the COD decreased to 495.3 and 405.3 mgO_2_ per L, corresponding to mineralization efficiencies of 30.8% and 43.6% for the two materials. Upon illumination, the COD continued to decrease rapidly while mineralization gradually increased. After six hours of illumination for 5CuH and five hours for 5CuH/Bent-2, the COD dropped to 82.0 and 88.7 mgO_2_ per L, with mineralization reaching 88.5% and 87.7%, respectively. These COD values met the allowable limit in Column B for a discharge flow rate ≤2000 m^3^ per day according to QCVN 40:2025/BTNMT – the National Technical Regulation on Industrial Wastewater (Vietnamese standard) (COD ≤ 90 mgO_2_ per L).

After five hours of illumination, the COD decreased sharply (from 262.0 to 98.7 mgO_2_ per L for 5CuH and from 215.3 to 88.7 mgO_2_ per L for 5CuH/Bent-2), likely due to the oxidation of dye molecules into low-molecular-weight intermediates that were more readily degraded. Extending the illumination time to nine hours further reduced the COD to 55.3 and 48.7 mgO_2_ per L for 5CuH and 5CuH/Bent-2, respectively. However, the mineralization rate slowed, reaching only 92.3% and 93.2%. In this investigation, 5CuH/Bent-2 demonstrated faster dye degradation and higher pollutant mineralization rates compared with 5CuH. These results confirmed that both 5CuH and 5CuH/Bent-2 exhibited high catalytic activity and mineralization efficiency, demonstrating their practical applicability for the treatment of textile wastewater.

#### 3.5.3. Practical implications

The studies conducted on dye removal and mineralisation showed that both 5Copper Hydroxyapatite (5CuH) and the composite 5CuH/bentonite-2 (5CuH/Bent-2) performed exceptionally well for treating actual textile wastewaters. The higher capacity for the adsorption of dye molecules, and the increased activity of the catalysts applying a combination of adsorption and photocatalysis, resulted in a faster rate of color removal and significantly higher rates of mineralisation with the 5CuH/Bent-2 relative to the 5CuH samples. Decolorization and mineralisation were achieved when the COD was brought below the maximum allowable level (≤90 mgO_2_ per L) within 5–6 hours of exposure to light, clearly demonstrating that this combination of materials is effective in reducing both the color and organic loading of textile wastewaters in practical applications.

The research has demonstrated that complete mineralisation of the contaminants will require additional time of exposure to light so as to avoid the accumulation of any potentially harmful intermediate by-products that may be produced during the process; therefore, enough time should be allowed for the safe and complete removal of contaminants from textile wastewaters as well.

From a practical standpoint, these materials have exhibited excellent levels of catalytic activity, long-term stability, and high potential for recycling, indicating that they will have a long service life in the textile wastewater treatment industry. The efficiencies of the 5CuH/Bent-2 in this regard, in addition to their cost-effective means of treating textile wastewaters (shortened period of time) and utilizing significantly less amounts of chemical additives than the treatment of textile wastewaters without these materials, create an incentive to continue further research into the application of CuH and Bent-2 for textile wastewater treatments. These findings indicate that the synthesised materials are not only effective under laboratory conditions but also have strong potential for scalable implementation in the textile industry.

### 3.6. Limitations and future perspectives

Despite the promising photocatalytic performance of the Cu(ii)-modified hydrotalcite–bentonite composites under visible light, several limitations of the present study should be acknowledged.

First, Cu-free layered double hydroxide materials and pristine Mg–Al hydrotalcite were not systematically evaluated as reference catalysts. Including these controls would provide clearer insight into the role of Cu species in activating the LDH structure under visible-light irradiation. Although previous studies report negligible visible-light activity for pristine hydrotalcite, future work should include systematic comparisons with Cu-free materials to better quantify the contribution of Cu incorporation.

Second, catalyst stability requires further investigation. A slight decrease in activity was observed during reuse experiments, and possible Cu leaching was not quantitatively examined in this work. Future studies should therefore include metal-leaching analysis and stability evaluation under varying pH, ionic strength, and ligand conditions to better assess long-term catalyst durability.

Third, Rhodamine B was used as a model pollutant, which cannot fully represent the complexity of real textile wastewater. Although preliminary experiments using actual wastewater were performed, more systematic selectivity studies involving mixed-dye systems and competing species are necessary to clarify degradation behavior in complex matrices.

Finally, process optimization remains necessary for practical application. The influence of catalyst dosage and temperature on photocatalytic efficiency was not systematically investigated, and future work should address these parameters to better evaluate the scalability and operational feasibility of the developed catalysts.

## 4. Conclusions

In this study, Cu(ii)-modified Mg–Al layered double hydroxide and its bentonite-supported composites were rationally designed *via* a facile co-precipitation strategy to construct visible-light-active photocatalysts for textile wastewater remediation. Structural analyses demonstrated that Cu incorporation not only narrowed the band gap and introduced a CuO phase but also modulated the electronic structure of the LDH framework, while bentonite integration enhanced dispersion and structural stability of active sites.

The optimized materials achieved >93% RhB degradation under visible-light irradiation and exhibited strong mineralization capacity, meeting national COD discharge standards. Mechanistic investigation confirmed that pollutant removal was predominantly governed by reactive oxygen species (˙OH and O_2_˙^−^) generated through improved charge carrier separation, rather than adsorption-driven processes.

The catalysts displayed good durability under repeated cycles and maintained high efficiency in real textile wastewater, demonstrating robustness in complex multicomponent environments.

Importantly, this work establishes a synergistic clay-LDH design principle that integrates electronic modulation and structural stabilization to enhance photocatalytic efficiency under visible light. The use of low-cost natural clay, simple synthesis, and demonstrated real-wastewater performance highlights the scalability and industrial relevance of this platform for sustainable advanced oxidation processes.

## Author contributions

Conceptualization: V. N. V.; methodology: V. N. V., N. T. T. L. and T. H. T. P.; software: T. T. H. P., T. T. L. N. and T. T. A. D.; validation: V. N. V., T. T. L. N. and T. T. H. P.; data curation: V. N. V., T. T. H. P. and T. T. A. D.; writing – original draft preparation, T. H. T. P., T. X. V. and V. N. V.; writing – review and editing, V. T. X., T. T. L. N.: visualization: V. N. V., T. X. V., and T. T. A. D. project administration, funding acquisition, resources: V. N. V. and T. H. T. P. All authors have read and agreed to the published version of the manuscript.

## Conflicts of interest

The authors declare no conflicts of interest.

## Supplementary Material

RA-016-D5RA09642A-s001

## Data Availability

All data obtained have been included in the manuscript, supplementary information (SI) and are available from the corresponding author upon reasonable request. Supplementary information: supporting data on material characterization and performance. Elemental compositions (Tables S1 and S2), band gap energies (Table S3 and Fig. S3), and calibration data (Fig. S1) are included. Adsorption and photocatalytic degradation efficiencies under various conditions (concentration, light, dark, and pH) are presented in Tables S4–S10. Scavenger experiments identifying reactive species are shown in Tables S11 and S12. The applicability to real wastewater is demonstrated through dye removal and COD reduction (Tables S13 and S14). Additional figures illustrate the experimental setup and visual changes during degradation (Fig. S2, S4 and S5). See DOI: https://doi.org/10.1039/d5ra09642a.
