# Supplementary material for: Cu(ii)-modified Mg–Al hydrotalcite/bentonite composites for adsorption and visible-light-driven photocatalytic degradation of Rhodamine B in textile wastewater
Source: RSC Adv. 2026 Mar 23;16(18):16001–29. doi: 10.1039/d5ra09642a (PMC13006885; doi:10.1039/d5ra09642a)
Supplement: RA-016-D5RA09642A-s001 [file RA-016-D5RA09642A-s001.pdf]

Supplementary Information (SI) for RSC Advances.
This journal is © The Royal Society of Chemistry 2026

SUPPLEMENTARY INFORMATION

Cu(II)-modified Mg–Al hydrotalcite/bentonite composites for adsorption and visible-light-driven photocatalytic degradation of Rhodamine B in textile wastewater

Table S1. Elemental composition of the 5CuH sample obtained from EDS analysis, expressed as weight percentage (wt%) and atomic percentage (at%).

STT	Element	% Weight	% Atom
1	**O**	45.77	69.95
2	**Mg**	5.48	5.52
3	**Al**	10.44	9.46
4	**C**	1.10	0.76
5	**Cu**	37.20	14.31
	**Sum**	100.00	

Table S2. Elemental composition of the 5CuH/Bent-2 sample obtained from EDS analysis, expressed as weight percentage (wt%) and atomic percentage (at%).

Nr	Element	% Weight	% Atom
1	**O**	48.91	70.17
2	**Mg**	4.15	3.92
3	**Al**	9.94	8.46
4	**Si**	8.51	6.96
5	**K**	0.31	0.18
6	**Ti**	0.29	0.14
7	**Fe**	1.97	0.81
8	**Cu**	25.93	9.37
	**Sum**	100.00	

Table S3. Optical band gap values of the synthesized materials

No.	Material	Band gap energy E_g (eV)
1	**Bent**	1.7; 2.5
2	**5CuH**	1.42
3	**5CuH/Bent-1**	1.97
4	**5CuH/Bent-2**	1.76
5	**5CuH/Bent-3**	2.12

Table S4. Adsorption efficiency of RhB (50 ppm) on different materials as a function of contact time

Adsorption time (min)	Average RhB removal efficiency (%)				
	Bent	5CuH	5CuH/Bent-1	5CuH/Bent-2	5CuH/Bent-3
**0**	0.0	0.0	0.0	0.0	0.0
**30**	36.4	2.8	2.4	2.9	2.1
**60**	35.9	2.6	2.3	1.8	0.9
**90**	34.2	2.8	1.8	1.5	0.9
**120**	33.5	2.1	1.7	1.5	0.8

Table S5. Removal efficiency of RhB (50 ppm) by the synthesized materials under visible light

**Time (min)**	**Average RhB removal efficiency (%)**					
	**Bent**	**5CuH**	**5CuH/Bent-1**	**5CuH/Bent-2**	**5CuH/Bent-3**	**Blank**
**0**	0.0	0.0	0.0	0.0	0.0	0.0
**30 ad**	35.5	2.8	2.5	2.7	2.2	
**60**	45.2	87.8	74.1	84.1	74.3	2.1
**90**	54.7	91.5	90.2	93.6	88.9	8.8
**120**	68.1	94.9	93.9	96.8	93.8	15.7
**150**	72.9	95.9	98.5	96.9	98.5	17.6
**180**	80.4	96.5	99.4	98.1	99.6	22.8

ad: adsorption; Blank: sample containing only H_2O_2 without materials.

Table S6. Removal efficiency of RhB (75 ppm) over the synthesized materials under visible light irradiation.

**Time (min)**	**Average RhB removal efficiency (%)**					
	**Bent**	**5CuH**	**5CuH/Bent-1**	**5CuH/Bent-2**	**5CuH/Bent-3**	**Blank sample**
**0**	0.0	0.0	0.0	0.0	0.0	0.0
**30(ad)**	29.1	2.7	2.3	2.6	2.2	0.0
**60**	43.0	72.4	52.2	65.8	53.5	5.8
**90**	48.8	89.6	73.2	86.2	71.1	8.8
**120**	61.8	95.6	85.6	94.5	82.3	8.6
**150**	68.7	98.1	92.6	98.1	90.9	11.3
**180**	72.2	98.9	97.1	98.7	95.4	14.8

ad: adsorption; the blank sample contained only H_2O_2 without catalyst.

Table S7. Removal efficiency of RhB (100 ppm) by the synthesized composite materials under visible light.

**Time (min)**	**Average RhB removal efficiency (%)**					
	**Bent**	**Blank**	**5CuH**	**5CuH/Bent-1**	**5CuH/Bent-2**	**5CuH/Bent-3**
0	0.0	0.0	0.0	0.0	0.0	0.0
30 (ad)	25.9	0.0	2.6	2.3	2.5	1.9
60	38.2	4.7	58.7	33.9	37.7	33.7
90	43.8	6.1	79.7	58.7	62.0	50.9
120	50.5	8.4	89.6	71.5	78.9	63.6
150	58.5	9.5	94.7	82.5	89.8	78.8
180	65.8	11.4	97.5	91.2	95.8	86.8
210	67.0	14.5	98.6	95.9	97.7	93.3

ad: adsorption; the blank sample contained only H_2O_2 without catalyst.

Table S8. RhB (75 ppm) removal efficiency under dark conditions using the synthesized materials

**Time (min)**	**Average RhB removal efficiency under dark conditions of materials (%)**					
	**Bent**	**5CuH**	**5CuH/Bent-1**	**5CuH/Bent-2**	**5CuH/Bent-3**	**Blank**
**0**	0.0	0.0	0.0	0.0	0.0	0.0
**30 ad**	26.2	2.8	2.5	2.6	2.2	0.0
**60**	32.2	38.6	17.3	20.8	19.3	1.6
**90**	34.1	63.5	24.5	40.7	36.3	3.4
**120**	36.8	78.5	44.3	61.9	50.5	4.1
**150**	39.4	87.2	59.4	75.6	64.7	5.6
**180**	41.2	90.8	66.6	85.1	73.8	7.5

ad: adsorption; the blank sample contained only H_2O_2 without catalyst.

Table S9. Removal efficiency of 5CuH toward 100 ppm RhB at different pH values

Time (min)	Average RhB removal efficiency at different pH values (%)					
	**pH = 3.0**	**pH = 3.9 (initial)**	**pH = 6.0**	**pH = 8.0**	**pH = 10.0**	**pH = 12.0**
0	0.00	0.00	0.00	0.00	0.00	0
30 (ad)	1.6	2.6	3.2	4.4	1.9	1.2
60	52.6	58.7	63.9	65.5	28.8	32.2
90	75.3	79.7	76.8	75.8	29.6	30.8
120	88.7	89.6	85.4	83.8	29.5	33.7
150	93.6	94.7	90.3	84.2	29.9	30.5
180	95.5	97.5	92.4	88.8	30.5	32.1
210	96.3	98.6	92.6	92.8	29.8	32.5

Table S10. Removal efficiency of 5CuH/Bent-2 toward 100 ppm RhB at different pH values

Time (min)	Average RhB removal efficiency at different pH values (%)					
	**pH = 3.0**	**pH = 3.9 (initial)**	**pH = 6.0**	**pH = 8.0**	**pH = 10.0**	**pH = 12.0**
**0**	0.0	0.0	0.0	0.0	0.0	0.0
**30 ad**	5.1	2.8	3.0	3.2	3.5	3.9
**60**	18.9	42.4	50.2	50.2	21.7	38.34
**90**	27.3	62.8	69.1	65.9	27.3	38.5
**120**	43.5	79.4	83.2	80.8	30.1	39.3
**150**	57.7	90.1	91.1	89.8	32.4	40.3
**180**	73.1	94.6	96.7	95.1	31.5	39.4
**210**	85.18	97.8	98.7	97.2	30.9	40.6

Table S11. Removal efficiency of 100 ppm RhB by 5CuH in the presence of electron, hole, and reactive oxygen species scavengers

**Time (min)**	**Average removal efficiency of 100 ppm RhB by 5CuH in the presence of different scavengers (%)**				
	**Ascorbic acid**	**EDTA**	**IPA**	**$AgNO_3$**	**5CuH**
**0**	0.0	0.0	0.0	0.00	0.0
**30 (ad)**	2.6	2.5	2.7	2.4	2.5
**60**	76.1	72.3	11.5	51.6	58.7
**90**	85.7	81.6	14.1	70.1	79.7
**120**	93.1	89.5	16.1	83.5	89.6
**150**	95.9	93.4	15.5	89.5	94.7
**180**	97.6	95.6	16.1	93.7	97.5
**210**	96.7	94.4	16.2	95.4	98.6

Note:5CuH refers to the sample without any scavenger present and (ad) refers to adsorption.

Table S12. Removal efficiency of 100 ppm RhB by 5CuH/Bent-2 in the presence of electron, hole, and reactive oxygen species scavengers.

**Time (min)**	**Average removal efficiency of 100 ppm RhB by 5CuH/Bent-2 in the presence of different scavengers (%)**				
	**Ascorbic acid**	**EDTA-2Na**	**IPA**	**$AgNO_3$**	**5CuH/Bent-2**
**0**	0.0	0.0	0.0	0.0	0.0
**30 (ad)**	2.5	2.6	2.5	2.7	2.8
**60**	79.5	80.6	11.8	53.2	37.7
**90**	89.5	89.8	17.3	72.5	62.4
**120**	95.1	95.4	18.3	86.7	78.9
**150**	98.2	98.2	18.4	93.7	89.8
**180**	99.5	99.5	20.2	97.9	95.8
**210**	99.4	99.8	22.2	99.2	97.6

Note:*5CuH/Bent-2 refers to the sample without any scavenger present and (ad) refers to adsorption.*

Table S13. Removal efficiency of dyes in mat-dyeing textile wastewater

**Time (min)**	**Removal efficiency of dyes in mat-dyeing textile wastewater (%)**	
	**5CuH**	**5CuH/Bent-2**
**0**	0	0
**60 (ad)**	37.9	58.2
**90**	61.9	64.8
**120**	65.8	70.7
**150**	68.1	73.3
**180**	74.6	74.6
**210**	74.9	77.6
**240**	83.7	86.1
**270**	90.2	91.2
**300**	91.0	92.3
**330**	95.6	96.3

(ad) refers to adsorption.

Table S14. Mineralization of dyes in mat-dyeing textile wastewater by 5CuH and 5CuH/Bent-2 as indicated by COD values at the initial pH (5.08)

**Time (hour)**	**COD (mgO_2/L)**		**Mineralization (%)**	
	**5CuH**	**5CuH/Bent-2**	**5CuH**	**5CuH/Bent-2**
**0**	715.3	718.7	0.0	0.0
**1 (ad)**	495.3	405.3	30.8	43.6
**2**	408.7	382.0	42.9	46.8
**3**	382.0	348.7	46.6	51.5
**4**	308.7	282.0	56.8	60.8
**5**	262.0	215.3	63.4	70.0
**6**	98.7	88.7	86.2	87.7
**7**	82.0	68.7	88.5	90.4
**8**	75.3	62.0	89.5	91.4
**9**	62.0	55.3	91.3	92.3
**10**	55.3	48.7	92.3	93.2

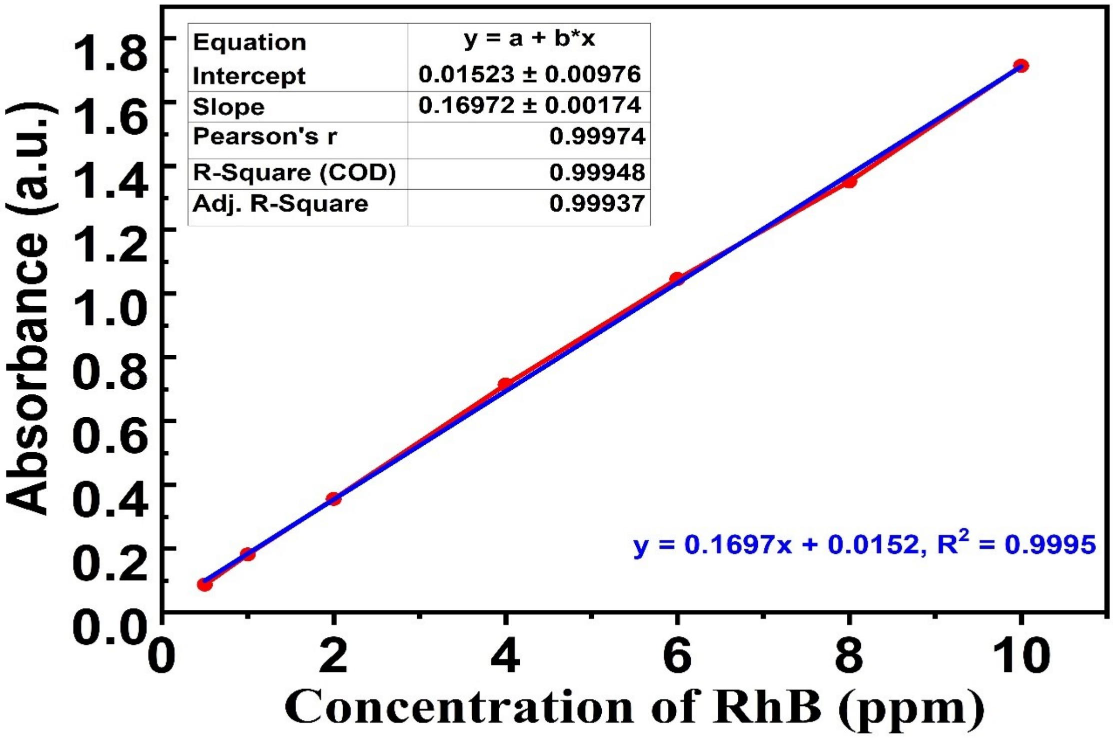

Figure S1. Plot showing the dependence of molecular absorbance on the concentration of RhB in solution (linear curve).

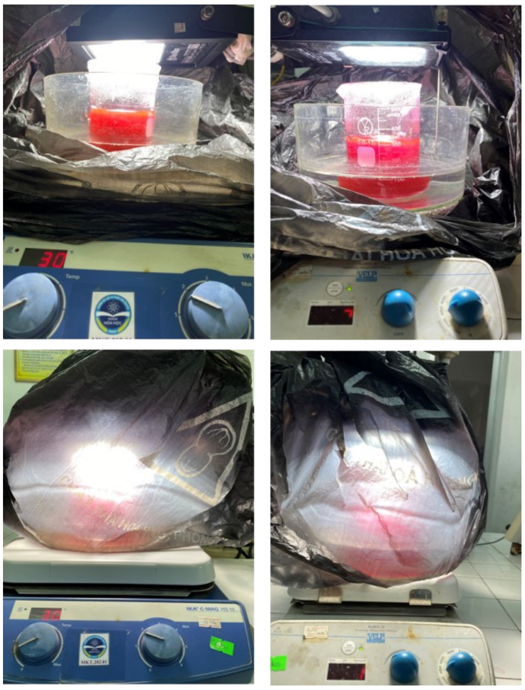

Figure S2. Experimental design for evaluating the photocatalytic degradation performance of RhB by the synthesized materials.

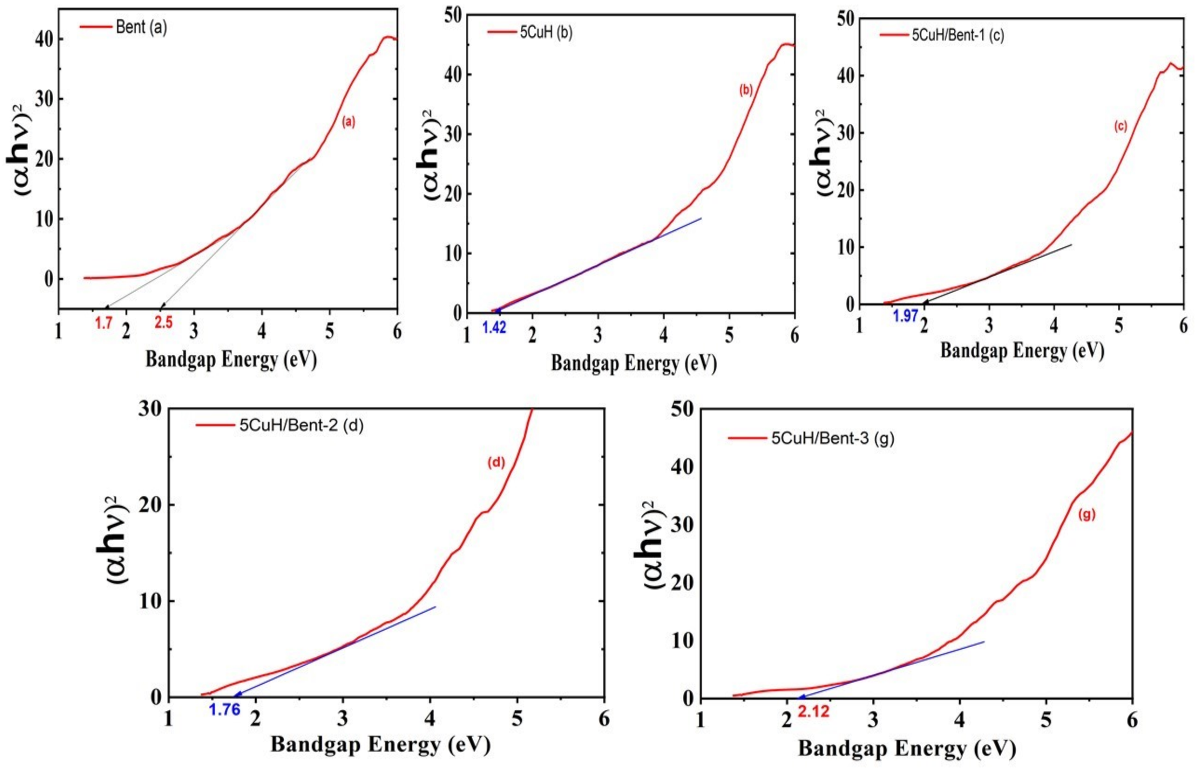

Figure S3. Optical band gap energies of the synthesized materials: Bent (a), 5CuH (b), 5CuH/Bent-1 (c), 5CuH/Bent-2 (d), and 5CuH/Bent-3 (g).

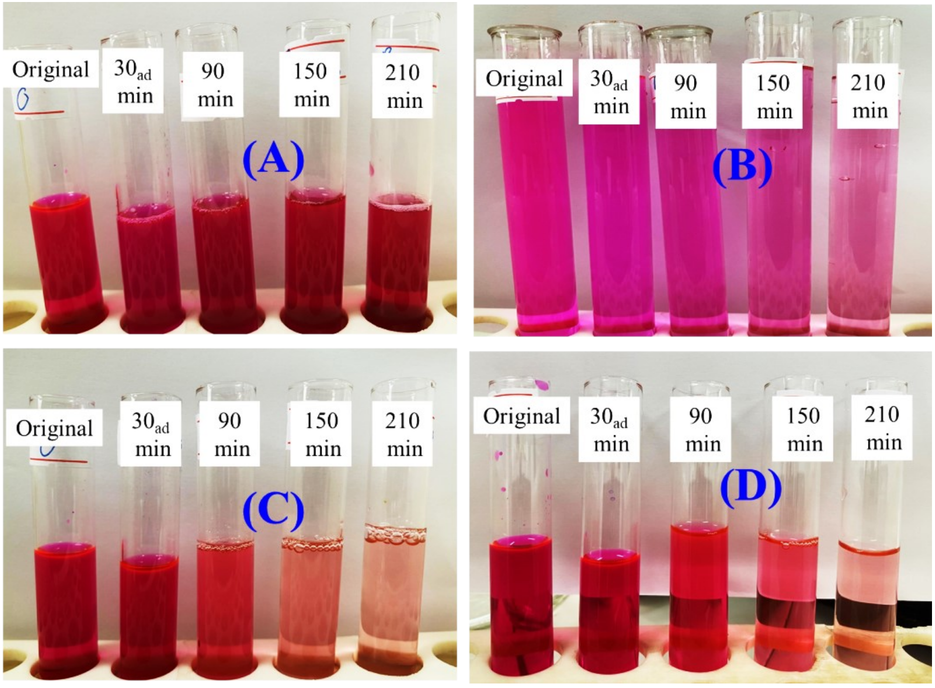

Figure S4. Color variation of RhB solution (initial concentration: 100 ppm) at different reaction times (initial, 30 min dark adsorption, 90, 150, and 210 min) over bentonite (A), 5CuH (C), and 5CuH/Bent-2 (D) samples (all solutions without dilution), and over bentonite (B) after 10-fold dilution.

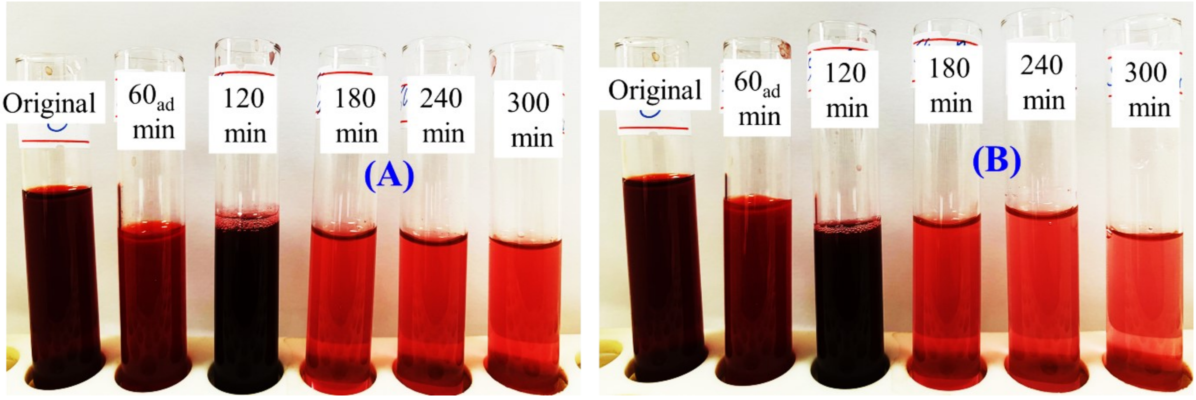

Figure S5. Color variation of woven-mat dyeing wastewater as a function of reaction time over 5CuH (A) and 5CuH/Bent-2 (B) samples at the initial stage, after 60 min of dark adsorption, and after 120, 180, 240, and 300 min of reaction (all solutions without dilution).
